# Flanking Bases Influence the Nature of DNA Distortion by Platinum 1,2-Intrastrand (GG) Cross-Links

**DOI:** 10.1371/journal.pone.0023582

**Published:** 2011-08-10

**Authors:** Debadeep Bhattacharyya, Srinivas Ramachandran, Shantanu Sharma, Wimal Pathmasiri, Candice L. King, Irene Baskerville-Abraham, Gunnar Boysen, James A. Swenberg, Sharon L. Campbell, Nikolay V. Dokholyan, Stephen G. Chaney

**Affiliations:** 1 Department of Biochemistry and Biophysics, School of Medicine, University of North Carolina, Chapel Hill, North Carolina, United States of America; 2 Program in Cellular and Molecular Biophysics, University of North Carolina, Chapel Hill, North Carolina, United States of America; 3 Department of Environmental Sciences and Engineering, School of Public Health, University of North Carolina, Chapel Hill, North Carolina, United States of America; 4 Lineberger Comprehensive Cancer Center, University of North Carolina, Chapel Hill, North Carolina, United States of America; Institut Pasteur, France

## Abstract

The differences in efficacy and molecular mechanisms of platinum anti-cancer drugs cisplatin (CP) and oxaliplatin (OX) are thought to be partially due to the differences in the DNA conformations of the CP and OX adducts that form on adjacent guanines on DNA, which in turn influence the binding of damage-recognition proteins that control downstream effects of the adducts. Here we report a comprehensive comparison of the structural distortion of DNA caused by CP and OX adducts in the TGGT sequence context using nuclear magnetic resonance (NMR) spectroscopy and molecular dynamics (MD) simulations. When compared to our previous studies in other sequence contexts, these structural studies help us understand the effect of the sequence context on the conformation of Pt-GG DNA adducts. We find that both the sequence context and the type of Pt-GG DNA adduct (CP vs. OX) play an important role in the conformation and the conformational dynamics of Pt-DNA adducts, possibly explaining their influence on the ability of many damage-recognition proteins to bind to Pt-DNA adducts.

## Introduction

Cisplatin (CP), carboplatin and oxaliplatin (OX) are platinum based drugs widely used in the treatment of many cancers [1]. The mode of action of CP and OX is through formation of adducts on genomic DNA, the most common of them being intra-strand Pt-GG adducts [2], [3], [4]. The main difference between CP and OX is in their carrier ligands: for CP it is diammine while for OX it is diaminocyclohexane. We [3] and others [2], [4] have shown that CP and OX form the same types of adducts (GG, AG, GNG and interstrand) at the same abundance and at the same sites on the DNA.

Cells and tumors resistant to CP are often not cross resistant to OX [5], [6], and many DNA damage recognition proteins that bind to Pt-GG adducts discriminate between CP- and OX-GG adducts [7], [8], [9], [10], [11], even though they form chemically similar adducts. The effectiveness of OX in CP-resistant cell lines is thought to be due to repair or damage-recognition processes that discriminate between CP and OX DNA adducts. This has been best established for mismatch repair. For example, the binding of the mismatch repair complex appears to increase the cytotoxicity of Pt-DNA adducts [8], [12], [13], [14], either by activating downstream signaling pathways that lead to apoptosis [15], [16], [17] or by causing “futile cycling” during translesion synthesis past Pt-DNA adducts [18]. These effects appear to be specific for CP adducts. Thus, defects in mismatch repair cause resistance to CP adducts [8], [12], [13], [19], but have no effect on cellular sensitivity to OX adducts [18]. As one might predict from these biological differences, hMSH2 [8] and MutS [9] bind with greater affinity to CP-GG DNA adducts than to OX-GG DNA adducts.

Some damage-recognition proteins such as HMGB1, LEF-1, TBP and hUBF also bind more tightly to CP-GG DNA adducts than to OX-GG DNA adducts [7], [10], [11], [20]. The biological consequences of these effects are less clear, but the binding of abundant chromatin architectural proteins like HMGB1 is thought to shield the adducts from nucleotide excision repair [21], [22], inhibit translesion synthesis [23] and/or initiate signaling pathways leading to cell cycle arrest or apoptosis [24]. The binding of low abundance transcription factors like hUBF to Pt-DNA adducts on the other hand is thought to sequester these transcription factors from their cognate binding sites on the genome [20], [25].

Of the damage-recognition proteins studied to date, the binding of HMGB1 to CP-DNA adducts has been characterized in the greatest detail. HMGB1 contains two HMG domains: domain A (HMGB1a) and domain B (HMGB1b). Footprinting studies combined with site-directed mutagenesis have shown that only domain A of full length HMGB1 binds to the portion of the DNA containing the CP-GG DNA adduct [26], [27]. Thus, most of the previous structural and mechanistic studies have been performed with HMGB1a alone. The strength of binding of HMGB1a to the CP-GG adduct and the ability of HMGB1a to discriminate between CP-GG and OX-GG adducts has been shown to be highly dependent on the sequence context of the Pt-GG adduct [7], [10], [28], [29].

The intercalation of an amino acid residue between two DNA bases, bending of the DNA in the direction of the major groove and protein-DNA interactions at the minor groove surface are a common feature of protein-DNA interaction by mismatch repair proteins [30], [31], [32], [33], HMG box proteins [34], [35] and several transcription factors [36], [37], [38], [39]. The binding of HMGB1 and other damage-recognition proteins to Pt-GG adducts is thought to be facilitated by the bend imposed in DNA by the formation of Pt-GG adduct. For example, the large positive roll and dihedral angle of the Pt-GG has been postulated to permit intercalation of an amino acid between the Gs, and the wide, shallow minor groove is thought to provide a suitable surface for protein binding [27], [40]. HMGB1 and other damage-recognition proteins that discriminate between CP-GG and OX-GG DNA adducts bind to the minor groove and never contact the drug, which is in the major groove. Thus, we have hypothesized that these damage-recognition proteins are recognizing structural distortions of the DNA that are caused by the Pt-GG adduct rather than recognizing the adducts themselves, and that the structural distortion caused by the formation of Pt-GG adducts is influenced by both the type of adduct (CP vs OX) and the sequence context of the adduct.

In order to characterize the distortion caused by the formation of Pt adducts, structures have been reported for CP-GG and OX-GG adducts in a number of different sequence contexts [41], [42], [43], [44], [45], [46], [47], [48], [49], [50]. The overall conformation of these Pt-DNA adducts appears to be similar. However, a detailed comparative analysis of their structural and conformational features has proven difficult as varying lengths of DNA have been used, and different techniques have been used to solve the Pt-DNA adduct structures. For example, NMR structures obtained to date have varied with respect to the number and resolution of NMR constraints obtained and the molecular mechanics simulations used to convert the NMR constraints to final structures [41], [45], [46], [47]. Thus, these structures have provided only limited insight into how damage-recognition proteins such as HMGB1 can discriminate between CP-GG and OX-GG or the effect of sequence context on the recognition of the adducts. It is also known that the conformational properties of neighboring dinucleotides in undamaged DNA can be influenced by sequence context and that this effect is particularly pronounced for GG dinucleotides [51]. Thus, it is also important to compare the Pt-DNA structures to undamaged DNA structures solved under the same conditions and in the same sequence context.

Previously, we have solved the NMR solution structure of CP-, OX- and undamaged DNA in the AGGC sequence context [45], [46] and have shown important differences in conformation of CP- and OX-DNA adducts. We have also used all-atom molecular dynamics (MD) simulations to show differences in conformational dynamics between CP- and OX-DNA adducts in the AGGC [52] and TGGA [53] sequence contexts and have shown that both the type of adducts (CP versus OX) and the sequence context of the Pt-GG intrastrand diadduct influence the conformational dynamics of DNA.

**Figure 1:**
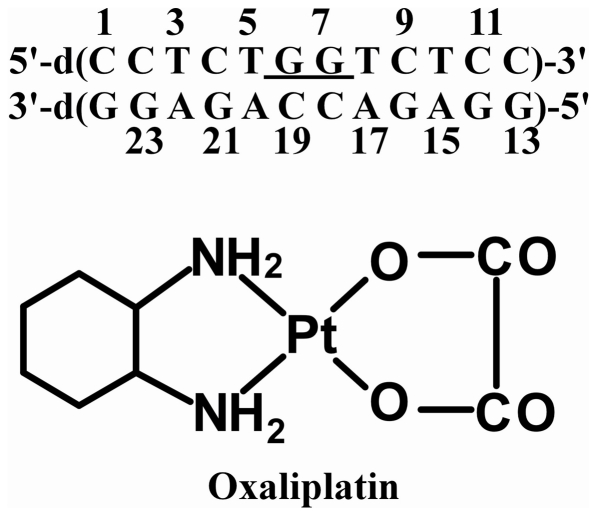
The duplex DNA sequence used in this study (top) and the chemical structure of oxaliplatin (bottom).

Our previous data have also shown that the NMR solution structures and the molecular dynamic simulations provide complementary insights into the structural differences that may be important for the differential recognition of CP- and OX-DNA adducts by various cellular proteins. Thus, in order to further understand the effects of carrier ligand and sequence context on Pt-DNA structure, we have solved high-resolution solution NMR structures of the OX-GG adduct and undamaged DNA duplex in the TGGT sequence context (Figure 1) and have performed molecular dynamics simulations of CP-, OX-GG adducts and undamaged DNA in the same sequence context. Because the NMR data were obtained using identical conditions and analysis methods with Pt-DNA adducts in both AGGC [45], [46] and TGGT sequence contexts, we were able to directly compare the effect of sequence context on the average DNA conformation in solution. Similarly, MD simulations on CP- and OX-DNA adducts have now been performed in an identical manner in three different sequence contexts which allows us to compare the effect of both carrier ligand and sequence context on the conformational dynamics of DNA. For example, combined with our earlier simulations in TGGA [53] and AGGC [52] sequence contexts, the current MD simulations allow us to ask whether the differences in conformational dynamics of CP-GG and OX-GG adducts in three different sequence contexts are consistent with the ability of proteins such as HMGB1a to discriminate between CP and OX adducts in all three sequence contexts.

## Results

### NMR characterization of the solution structure of the OX-GG adduct and undamaged DNA in the TGGT sequence context

**Figure 2:**
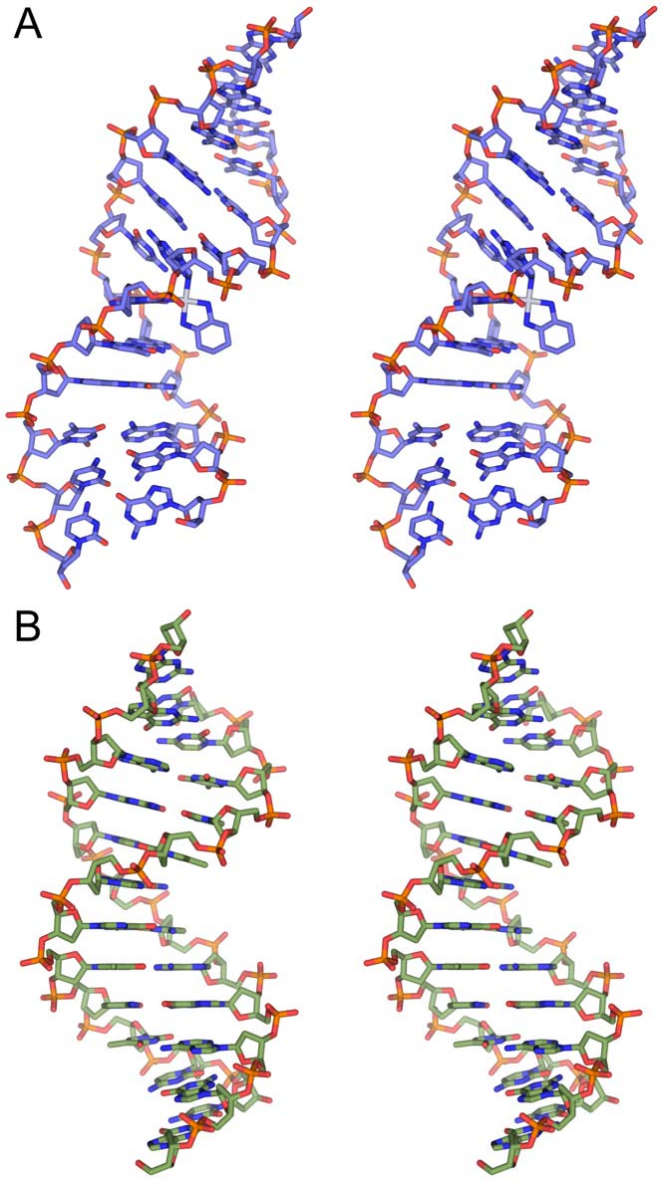
Stereo view of OX-TGGT and undamaged DNA TGGT solution structures. Lowest energy structure calculated for OX-DNA (**A**) and undamaged DNA (**B**) in the TGGT sequence context are displayed using stick representation, generated in PyMOL (www.pymol.org).

The 12-mer oligonucleotide containing the TGGT sequence (Figure 1) was platinated according to our previously described protocols and purified using HPLC (described in Methods). Collection of NOESY and 2D DQF-COSY spectra (Figures S1 and S2), and chemical shift assignments for OX-DNA and undamaged DNA duplex in the TGGT sequence context (Tables S1 and S2) were performed essentially as described previously for the OX-DNA, CP-DNA adducts and undamaged DNA in the AGGC sequence context [45], [46] (see Methods). The average solution structures of OX-DNA and undamaged DNA in the TGGT sequence context were computed from the NMR data essentially as described previously for OX-DNA, CP-DNA and undamaged DNA in the AGGC sequence context [45], [46] (details given in Methods. Coordinates and NMR restraints of the structures of OX-TGGT adduct and undamaged DNA have been deposited in the Protein Data Bank with accession numbers 2k0t (13 lowest energy structures of OX-DNA), 2k0u (average structure of OX-DNA calculated from the 20 lowest energy structures) and 2k0v (average structure of undamaged DNA calculated from the 20 lowest energy structures)). The stereo view of the average solution structures of the OX-TGGT adduct and undamaged TGGT DNA duplex are shown in Figure 2.

### Comparison of undamaged DNA and OX-GG adducts in the TGGT and AGGC sequence contexts

**Figure 3:**
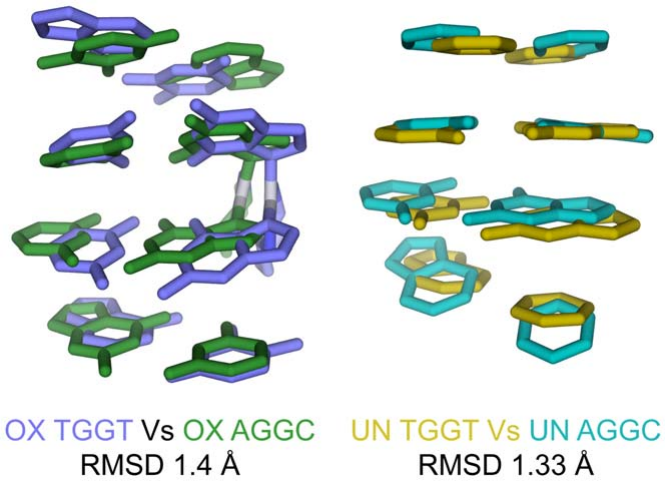
Comparison of OX-GG and undamaged DNA solution structures in the TGGT and AGGC sequence contexts. Structural alignment of OX-DNA in the TGGT and AGGC sequence context (left) and undamaged DNA in the TGGT and AGGC sequence context (right) are displayed using stick representation, generated in PyMOL (www.pymol.org). The DNA atoms common to AGGC and TGGT sequence context was used in the structural alignment of the central four base-pairs.

The similarity in the experimental procedures followed in this study and our previous work with CP-GG adducts, OX-GG adducts and undamaged DNA in the AGGC sequence context [45], [46] allow us to perform a systematic comparison of the key structural features of OX-GG adducts and undamaged DNA in two different sequence contexts. Overlays of the OX-TGGT adduct and undamaged TGGT DNA duplex with the corresponding structures in the AGGC sequence context are shown in Figure 3. Based on the RMSD values, sequence context (TGGT versus AGGC) has no major effect on the overall conformation of either undamaged DNA or OX-GG DNA adducts.

**Figure 4:**
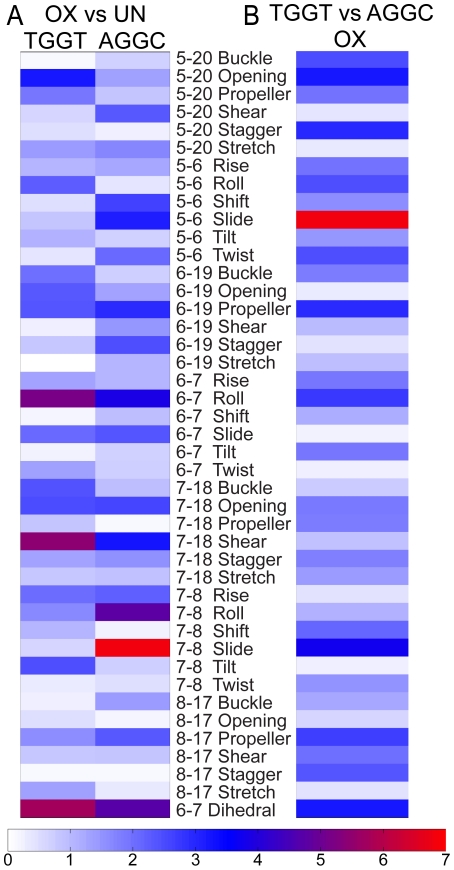
Conformational differences between the NMR structures of OX-DNA and undamaged DNA in the TGGT and AGGC sequence contexts. Heat maps of the Z-scores from comparisons of the 14 lowest energy NMR structures of OX-DNA and undamaged DNA in the TGGT and AGGC sequence contexts (A), and Z-scores of comparisons between the OX-TGGT and OX-AGGC DNA adducts (B).

In order to probe the possibility that more subtle conformational differences between these DNA structures might exist we employed CURVES version 5.3 [54] to calculate the helical parameters for the central four base-pairs and the central two base-pair steps of our OX-GG and undamaged DNA NMR solution structures (Table S3). We then identified the helical parameters that showed statistically significant differences between distributions of the 14 lowest energy structures for each NMR data set (see Methods) on the basis of their Z-score [55] (described in Text S1). Pair-wise comparisons of OX-DNA and undamaged DNA in the TGGT and AGGC sequence contexts are shown in Figure 4 as a heat map of Z-scores (described in Text S1). The heat maps allowed rapid identification of the helical parameters that differ the most between any two structures, while the DNA helical parameters themselves (Table S3) provide a description of the conformational differences between the structures.

Figure 4A and Table S3 show the comparison of the OX-GG adducts with undamaged DNA in both the TGGT and AGGC sequence contexts. In both sequence contexts, the formation of the OX-GG adduct resulted in a significant increase in roll and dihedral angle at the G6–G7 base pair step compared to undamaged DNA (Figure 4A, Table S3). This is a common feature of all Pt-GG structures reported to date [41], [42], [43], [44], [45], [46], [47], [48], [49], [50] and is thought to be important for the recognition of Pt-GG DNA adducts by HMGB1 and other damage-recognition proteins (see Discussion).

The OX-TGGT adduct is also similar to all other Pt-GG adducts with respect to a significant changes of DNA helical parameters of bases on both the 5′ and 3′ side of the adduct compared to undamaged DNA (Figure 4A, Table S3). However, the exact nature of these distortions appears to be dependent on the sequence context of the adduct. For example, the base pair step on the 5′ side of the OX-TGGT adduct differs from undamaged TGGT DNA primarily in terms of a large negative roll, while the base pair step on the 3′ side the OX-TGGT adduct differs from undamaged TGGT DNA primarily in terms of tilt (Figure 4A, Table S3). In contrast, the base pair step on the 5′ side of the OX-AGGC adduct differed from undamaged DNA primarily in terms of a negative shift and slide, while on the 3′ side, the OX-AGGC adduct differed from undamaged AGGC DNA in primarily in terms of and slide (Figure 4A, Table S3).

Another approach to analyzing the sequence context effects on conformation is to compare the conformations of the OX-TGGT and OX-AGGC adducts directly without reference to the corresponding undamaged DNA structures (Figure 4B). In this comparison, the most significant conformational differences appear to be the slide of the base pair steps on both the 5′ and 3′ side of the OX-GG adduct. Because DNA sequence can influence the conformation of undamaged DNA also [51], we also compared undamaged DNA in the TGGT and AGGC sequence contexts (data not shown). While some conformational differences were apparent for undamaged DNA in the two sequence contexts (Table S3), they were not large enough to influence the direct comparison of OX-TGGT and OX-AGGC adducts. Thus, these data show that the conformational distortion on the 5′ and 3′ side of OX-GG adducts is significantly affected by the sequence context of the adduct (TGGT versus AGGC), which may be of importance in understanding the sequence specificity of recognition of Pt-DNA adducts by HMGB1 and other damage-recognition proteins (see Discussion).

### Sequence context affects the conformational flexibility on the 5′ side of the adduct

**Figure 5:**
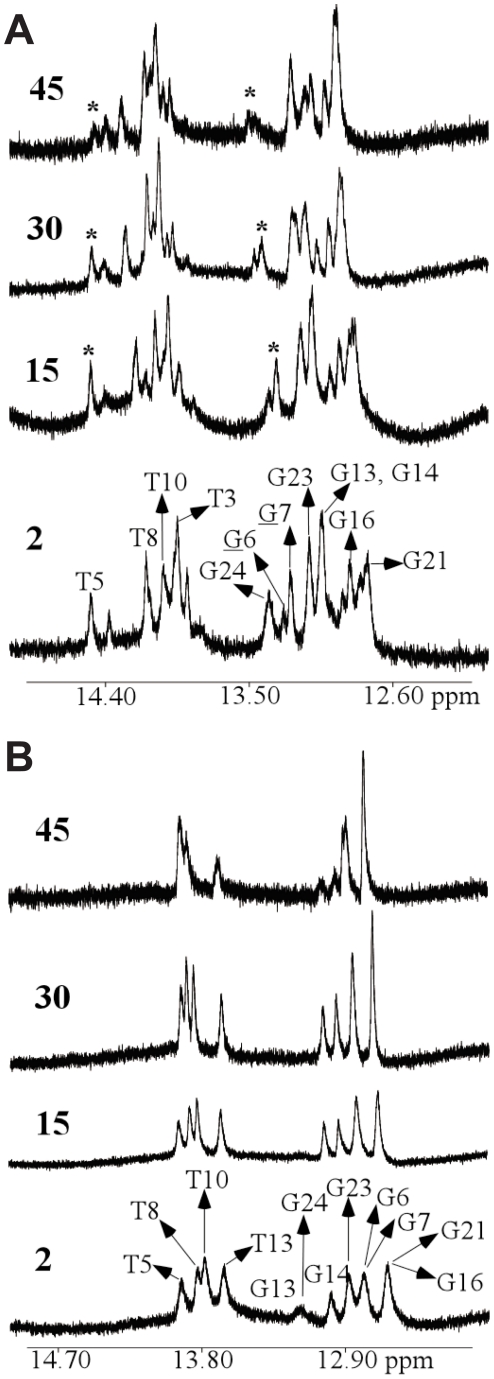
Temperature dependence of the imino proton signals by 1D NMR. Expanded imino region from 1D ^1^H-NMR spectra of the OX-DNA (**A**) and undamaged DNA (**B**) duplexes recorded in an H_2_O buffer at various temperatures (°C). The positions of the nucleotides in the 12-mer duplexes that give rise to the resonances are indicated. The asterisk (*) in the 1D ^1^H-NMR spectra of the OX-DNA adduct indicates the position(s) of the G6, G7, and T5 residues. The experimental temperatures are shown on the left. Also see Tables S1, S2 and S3.

The temperature dependence of the imino proton resonances of OX-GG adduct and undamaged DNA duplex (Figure 5) was monitored by 1D ^1^H-NMR in H_2_O buffer solution (100 mM NaCl, 5 mM Na_2_HPO_4_/NaH_2_PO_4_, pH 7.0) varying the temperature from 2°C to 45°C. For the OX-GG adduct in the TGGT sequence context, the imino signals of G6 and G7 disappear with increasing temperature while other imino peaks are observed even at 45°C. Moreover, the G6 imino peak for the OX-GG adduct in the TGGT sequence context shows a slightly faster solvent exchange rate relative to the G7 imino peak. These observations are consistent with previous reports for other Pt-GG adducts [41], [44], [45], [46], [47], and indicate that DNA containing both the CP and OX adducts is more solvent accessible on the 5′ side of the adduct than on the 3′ side, which suggests that the DNA may be more distorted and/or flexible on the 5′ side of the adduct.

In addition to the higher solvent exchange rate exhibited by the G6 and G7 imino protons, the T5 imino proton also displayed a fast exchange rate, which is comparable to the exchange rate shown by the G6 and G7 imino protons (Figure 5A). This feature was not observed for undamaged DNA (Figure 5B). A similar rapid exchange rate has previously been reported for the 5′T imino proton of a CP-TGGT DNA duplex [44]. The temperature dependence of the imino proton signals have previously been reported for CP-GG adducts in the CGGC sequence context [47] and for both CP- and OX-GG adducts in the AGGC sequence context [45], [46]. While the 5′ flanking bases do not possess imino proton signals, their complementary bases (G and T) in the opposing strand do possess imino signals; and no loss of signal from their imino protons was observed at increasing temperature. The fast solvent exchange rate seen for T in the 5′ T•A base pair, but not for either T in the 5′A•T base pair or G in the 5′ C•G base pair suggests that the 5′ T•A base pair in the TGGT sequence context is more solvent accessible than either the 5′ A•T or 5′ C•G base pairs in the AGGC and the CGGC sequence contexts. We hypothesize that the distortion and/or flexibility observed on the 5′- side of the Pt-GG adduct is extended to the 5′-flanking residue base-pair in the TGGT sequence context but not in the AGGC or CGGC sequence contexts. This difference in conformational flexibility of the 5′-flanking residue is consistent with the molecular dynamics simulations described in the next section and could influence the sequence context specificity of protein recognition of Pt-GG adducts (see Discussion).

### Molecular dynamics simulations: Conformational dynamics of CP-, OX- and undamaged DNA in the TGGT sequence context

**Figure 6:**
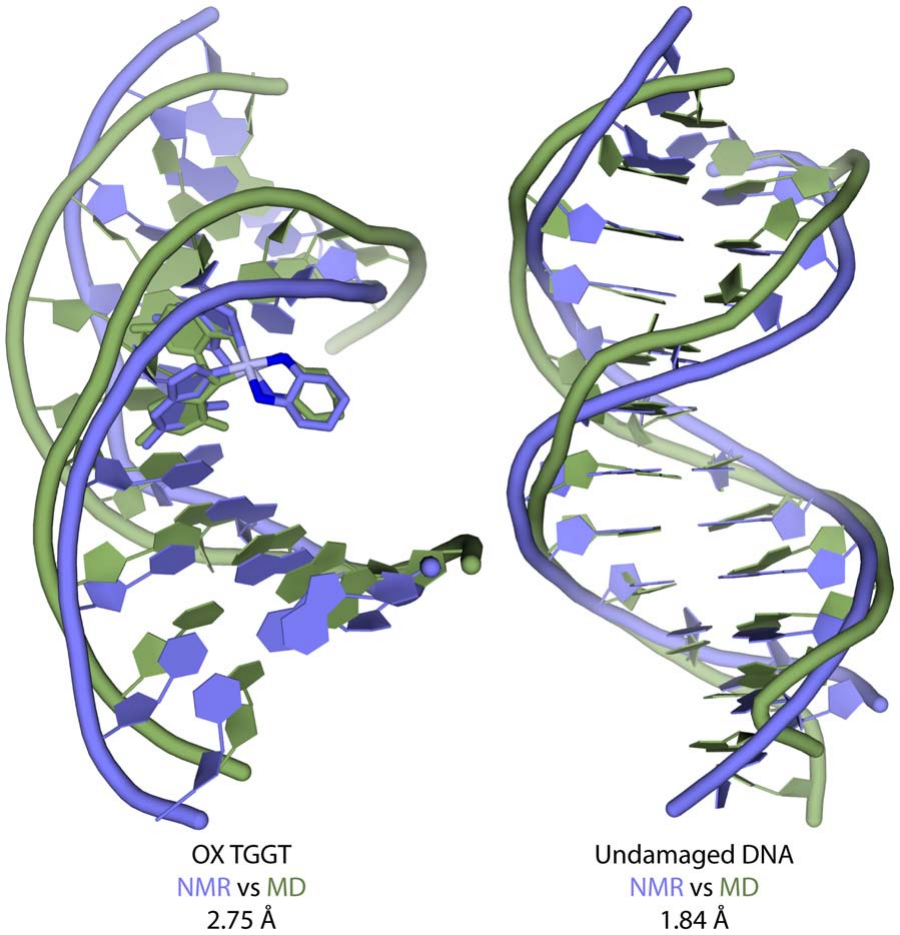
Comparison of NMR average structures and MD centroid structures. Structural alignment of the NMR average structures and MD centroid structures using only the atoms from the DNA part of OX-DNA (left) and undamaged DNA (right).

In an effort to better understand the effect of sequence context on the conformational dynamics of Pt-DNA adducts, we performed multiple 10 ns all-atom MD simulations (see Methods for details) of CP-, OX-GG adducts and undamaged DNA in the TGGT sequence context and compared them to our earlier simulations in the AGGC and TGGA sequence contexts [52], [53]. The simulations attained equilibrium within the first few nanoseconds, with the all-atom mass weighted RMSD of undamaged DNA remaining less than 3 Å and the RMSD of CP-DNA and OX-DNA remaining less than 4 Å to the starting structure throughout the simulation (Figure S3). The equilibrium structures were independent of the starting structure and initial velocities indicating that the simulations were well equilibrated. The centroid structures determined from the undamaged DNA and the OX-DNA simulations had RMSDs of 1.8 and 2.8 Å relative to their corresponding NMR structures (Figure 6). Additional evidence that the MD simulations were congruent with the NMR structures is included in Supporting Data Text S1.

### Sequence context dependent effects of OX-GG adducts as inferred from conformational dynamics

To explore the differences in conformational dynamics between the MD simulations for each structure, we calculated the helical parameters of the central four base-pairs and the central three base-pair steps for the ensembles of all the snapshots from each of the MD simulations using CURVES version 5.3 [56], [57]. We then compared the conformational dynamics of these simulations by constructing histograms of the helical parameters that showed statistically significant differences between distributions of any two ensembles (Figure S4) as determined using Kolmogorov-Smirnov (KS) ratio [55] (Text S1). From these comparisons two distinct effects of sequence context were observed. In the first case, formation of the OX-AGGC adduct, but not the OX-TGGT adduct, induced a significant change in conformational dynamics compared to undamaged DNA (Figure S4, Panels A and B), which tended to occur primarily on the 5′ side of the adduct. In the second case, formation of the OX-TGGT adduct, but not the OX-AGGC adduct induced a significant change in the conformational dynamics compared to undamaged DNA (Figure S4, Panels C and D). These differences in conformational dynamics were more subtle and tended to occur on the 3′ side of the adduct. The inclusion of undamaged DNA in the analysis was valuable because it allowed us to exclude differences in conformational dynamics between the OX-TGGT and OX-AGGC adducts that were primarily due to the effect of sequence context on undamaged DNA (Figure S4, Panels E and F).

### Hydrogen bond formation between Pt-amines and adjacent bases

**Figure 7:**
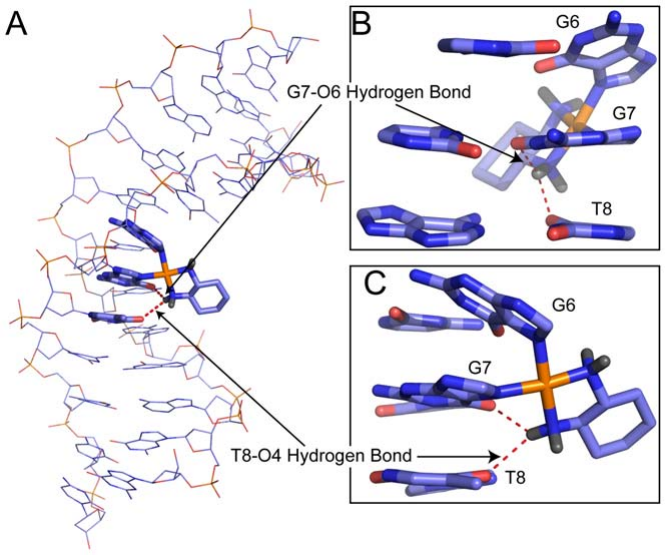
Hydrogen bond formation between the platinum amines and adjacent bases in OX-TGGT DNA adducts. Representative structure of OX-DNA in the TGGT sequence context that forms both the G7-O6 hydrogen bond and the T8-O4 hydrogen bond is shown using line representation (**A**) in PyMOL (www.pymol.org). The dach ligand, G6, G7 and A8 bases are shown using stick representation. The dach ligand, G6, G7 and A8 base-pairs of the same structure are shown in greater detail with the minor groove facing the reader (**B**) and along the side (**C**).

We have previously reported the formation of hydrogen bonds between Pt-amines and adjacent bases in our simulations of CP-DNA and OX-DNA adducts in both the AGGC and TGGA sequence contexts [52], [53]. We see a similar occurrence on the 3′ side of CP-DNA and OX-DNA adducts the TGGT sequence context, with hydrogen bonds observed between the 3′ Pt-amine and either the O6 atom of the 3′ Guanine (the G7-O6 hydrogen bond) or the O4 atom of 3′ Thymine (the T8-O4 hydrogen bond) (Figure 7). No significant hydrogen bond formation was observed between the Pt-amines and bases on the 5′ side of the CP- or OX-TGGT adducts. Even though similar hydrogen bonds are observed with both CP- TGGT and OX-TGGT DNA, the frequency of hydrogen bond formation is different for the CP- and OX-DNA adducts. The G7-O6 hydrogen bond is formed more frequently by OX-DNA (72% of the time compared to 32% for CP-DNA, Table 1), while the T8-O4 hydrogen bond is formed more frequently by CP-DNA (49% compared to 19% for OX-DNA).

**Table 1:** Distribution of hydrogen bonds between Pt-amines and adjacent bases observed in MD simulations.

TGGT			
CP		OX^a^	
Hydrogen bond type	Frequency	Hydrogen bond type	Frequency
G7-O6	20%	G7-O6	59%
T8-O4	37%	T8-O4	6%
T8-O4+G7-O6	12%	T8-O4+G7-O6	13%
None	29%	None	20%

AGGC^b^			
CP		OX^c^	
G7-O6	13%	G7-O6	34%
A5-N7	40%	A5-N7	14%
A5-N7+G7-O6	34%	A5-N7+G7-O6	45%
None	13%	None	8%

a Only the equatorial hydrogen of the OX-amine was involved in hydrogen bonding on the 3′ side of the adduct.

b from (Sharma *et al.*, 2007[52]).

c Both equatorial and axial hydrogens of the OX-amine were involved in hydrogen bonds on the 5′ side, and only equatorial hydrogen of the OX-amine was involved in hydrogen bonding on the 3′ side of the adduct.

### Conformational differences between species forming different hydrogen bonds between Pt-amines and adjacent bases

The characterization of the hydrogen bonds between platinum amines and the adjacent bases is of importance because each of those hydrogen bonds is associated with minor DNA conformations that may influence protein recognition of Pt-DNA adducts [52]. For example, we have shown that minor DNA conformations associated with hydrogen bond formation between the platinum amines and adjacent bases were consistent with the slight preferential recognition of CP-GG adducts by HMGB1a in the AGGC sequence context [52] and the very strong preferential recognition of CP-GG adducts by HMGB1a in the TGGA sequence context [53].

**Figure 8:**
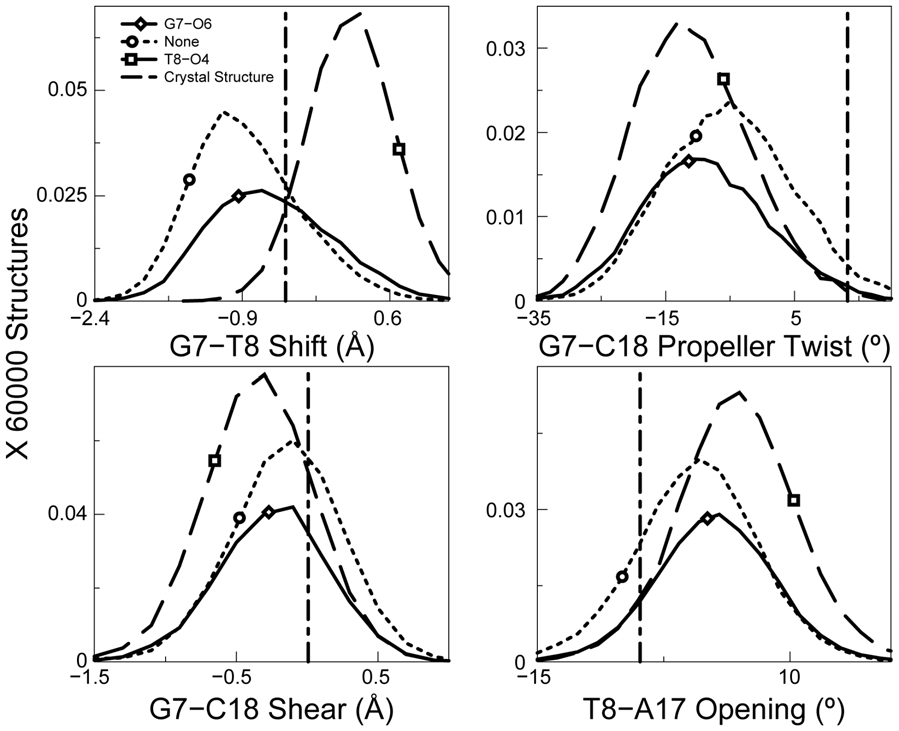
Helical parameters in the CP-TGGT sequence context. Four helical parameters plotted as a histogram for CP-TGGT. The most significant differences for different hydrogen bonded species of CP-DNA adduct are shown. The normalization was performed over the full 60000 structures to show the relative abundance of different hydrogen bonded species. The distribution for structures with no hydrogen bond formation is plotted in dotted lines, structures with G7-O6 hydrogen bond are designated by a solid line and structures containing a T8-O4 hydrogen bond are plotted in dashed line. The corresponding helical parameters for DNA in the crystal structure of CP-DNA bound to HMGB1a are plotted as a vertical dashed line.

**Figure 9:**
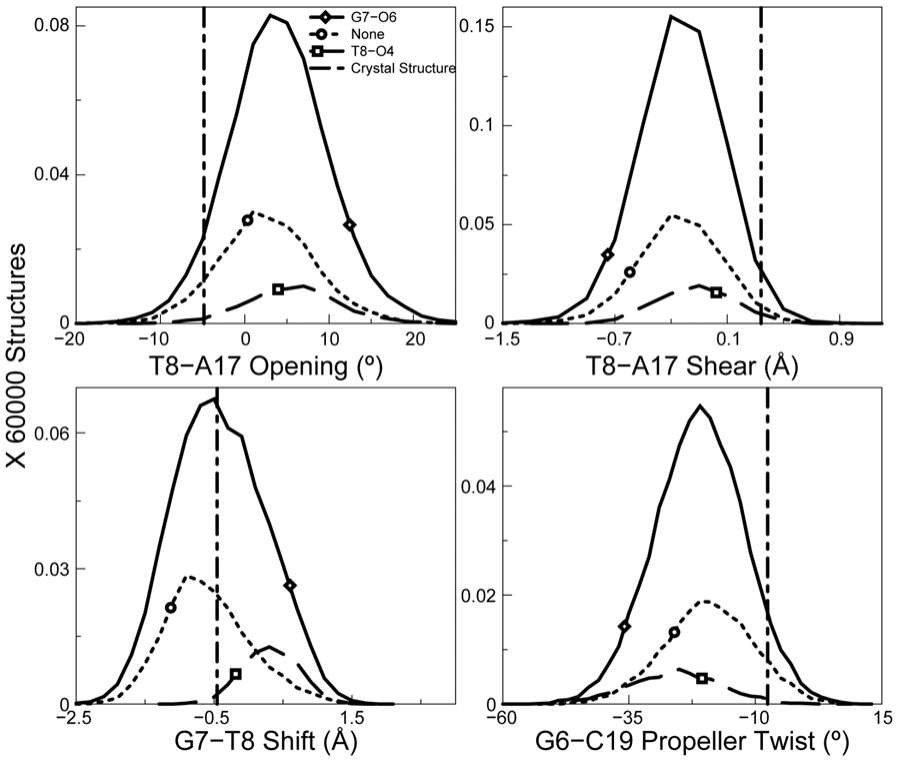
Helical parameters in the OX-TGGT sequence context. Four helical parameters plotted as a histogram showing the most significant differences for different hydrogen bonded species of the OX-DNA adduct are shown. The frequency distributions were calculated in a manner identical to that described in Figure 8. The distribution for structures with no hydrogen bond is plotted in dotted lines, for structures with G7-O6 hydrogen bond plotted with a solid line and structures with T8-O4 hydrogen bond, is plotted in dashed line. The bend angle of DNA in the crystal structure of CP-DNA bound to HMGB1a is plotted as a vertical dashed line.

Therefore, in the TGGT sequence context, we clustered the structures according to those forming the G7-O6 hydrogen bond, the T8-O4 hydrogen bond and those forming no hydrogen bonds and determined the helical parameters for each of those structures using CURVES version 5.3 (see Methods). As described previously, we then identified the helical parameters that showed statistically significant differences between distributions of any two ensembles on the basis of heat maps (Figure S5) of their Kolmogorov-Smirnov (KS) ratios [55] (described in Text S1). We observed no major differences in conformation between structures forming the G7-O6 hydrogen bond and those forming no hydrogen bonds for both CP- and OX-DNA adducts (data not shown). We had observed a similar result in the TGGA sequence context [53]. These data suggest that the formation of a hydrogen bond between the 3′ Pt-amine and G7-O6 does not require significant distortion of the Pt-GG adduct. However, in the CP-TGGT adducts, we found significant differences in the helical parameters between structures forming the T8-O4 hydrogen bonds and those forming either the G7-O6 hydrogen bond or no hydrogen bonds (Figure S5, panel A and Figure 8). For example, we observe more positive values for shift of the G7-T8 base-pair step and T8-A17 opening and more negative values for G7-C18 propeller twist and G7-C18 shear, which promote formation of the T8-O4 hydrogen bond. In OX-TGGT adducts, we observed shifts in helical parameters similar to CP-TGGT adducts for the structures forming the T8-O4 hydrogen bond (Figure S5 panel B and Figure 9). However, these effects on T8-O4 hydrogen bond formation are overshadowed by the heterogeneous distribution of structures forming the G7-O6 hydrogen bond. In the case of the Pt-TGGT adducts, the differences in conformation associated with the formation of the T8-O4 hydrogen bond do not appear to favor formation of the CP-GG-HMGB1a complex based on the comparison of the conformational distributions associated with formation of G7-O6 and T8-O4 hydrogen bond with the conformation observed in the crystal structure of the CP-GG-HMGB1a complex (the vertical line in Figures 8 and 9). These data are consistent with the very limited ability of HMGB1a to discriminate between CP-GG and OX-GG DNA adducts in the TGGT sequence context (see Discussion).

## Discussion

The recognition of Pt-GG DNA adducts by damage-recognition proteins, mismatch repair proteins and translesion DNA polymerases depends on both the nature of the Pt-GG adduct (CP versus OX) [7], [8], [9], [10], [11] and the sequence specificity of the DNA immediately surrounding the adduct [7], [10], [28], [29]. To better understand the mechanism(s) behind this differential protein-DNA binding, we have applied NMR and MD simulations as complementary methods to investigate the conformation and conformational dynamics of CP- and OX-GG DNA adducts in various sequence contexts. The NMR data provide information on the differences in average conformations of these adducts, while MD offers insight into the conformational dynamics and the existence of minor conformations. Because we have utilized identical experimental and computational approaches to solve the solution structures of CP-AGGC [58], OX-AGGC [46] and OX-TGGT adducts; and identical computational approaches to perform the MD simulations of CP-GG and OX-GG adducts in the TGGT, AGGC [52] and TGGA [53] sequence contexts, we feel that we are in a position to make detailed comparisons of these structures.

We have also evaluated the significance of the conformational differences that we observed by comparing them with the known binding specificity of HMGB1a for Pt-GG adducts [7], [10], [11], [28], [29]. We have chosen the specificity of HMGB1a binding as a criterion for evaluating the predictive value of our structures because the crystal structure of HMGB1a in complex with a CP-GG adduct is available [40], site-directed mutagenesis experiments have defined the effect of individual amino acid-DNA interactions on the strength of binding [27] and the effect of DNA sequence context on binding of HMGB1a to both CP-GG and OX-GG adducts has been defined in great detail [7], [10], [59].

For example, binding experiments have shown that the relative affinity of HMGB1a for Pt-GG adducts is generally Pt-TGGA > Pt-TGGT > Pt-AGGC [28], [59]. In addition, HMGB1a generally binds to CP-GG adducts with greater affinity than to OX-GG adducts, and the ability of HMGB1a to discriminate between CP- and OX-GG adducts is affected by sequence context in the order of Pt-TGGA > Pt-AGGC > Pt-TGGT [7], [10], [28], [59]. In fact, for Pt-TGGT adducts, there is little or no discrimination by HMGB1a between CP- and OX-GG adducts [7], [10].

The crystal structure of the HMGB1a-CP-GG DNA complex [40] shows that binding of HMGB1a is characterized by Phe37 intercalation between the two Gs of the Pt-GG adduct, Ser41 hydrogen bonding to the residue on the 3′ side of the Pt-GG adduct and multiple hydrophobic and electrostatic interactions between amino acid residues on the protein and the minor groove of the DNA. Site-directed mutagenesis experiments have shown that the Phe37 intercalation has by far the strongest effect on HMGB1a binding to the Pt-GG adduct [26], [27] and it has been postulated that the large positive roll and dihedral angle imposed on the two Gs of the Pt-GG adduct facilitate the intercalation of Phe37 between the central Gs [26], [27], [40].

Our NMR experiments have allowed a direct comparison of the CP-AGGC [58], OX-AGGC [46] and OX-TGGT (this study) adducts, along with undamaged DNA in the AGGC and TGGT sequence contexts ([58], this study). It has been postulated that the imposition of a large positive roll and dihedral angle on the two Gs of the Pt-GG adduct facilitates the binding of HMGB1a to the Pt-GG adduct [26], [27], [40]. When one compares the roll and dihedral angle for our three Pt-GG NMR structures ([58], Table S3), they are in the order of OX-TGGT > CP-AGGC > OX-AGGC, which correlates with the binding specificity of HMGB1a for these adducts [7], [10], [28], [59].

In addition, we have reported a greater solvent exchange rate for the imino proton on the 5′ side of the OX-TGGT adduct (Figure 5) than for either the OX-AGGC adduct [46] or the CP-AGGC adduct [58]. Yang *et al.* [44] have reported a similar rapid solvent exchange rate for the same imino proton on the 5′ side of the CP-TGGT adduct. We postulate that the greater solvent exchange rate indicates a greater conformational flexibility on the 5′ side of Pt-TGGT adducts than Pt-AGGC adducts. Since binding of HMGB1a to Pt-GG adducts requires a significant increase in both the roll and the dihedral angle of the central Gs [40], this increased conformational flexibility could also favor binding of HMGB1a to Pt-TGGT adducts.

We have also shown that the NMR solution structures of OX-AGGC and OX-TGGT adducts differ in the nature of the distortions imposed on both the 5′ and 3′ side of the adduct (Figure 4, Table S3). Additionally, when comparing the overall conformational flexibility of OX-TGGT and OX-AGGC adducts in our MD simulations (Figure S5), it was again apparent that the most significant differences between the OX-TGGT and OX-AGGC adducts were on the 5′ and 3′ sides of the adduct. However, when the average conformations (NMR data) and the range of conformations (MD data) for each helical parameter were compared with the conformation of DNA in the HMGB1a-CP-GG crystal structure [40], there was no clear correlation between differences observed on the 5′ and 3′ side of TGGT and AGGC adducts and the conformation of the HMGB1a-CP-GG complex (data not shown). Thus, while these conformational differences could influence the sequence specificity of HMGB1a binding, the mechanism by which this might occur is not clear.

In this and our previous MD simulations of Pt-GG adducts, we have shown the formation of transient hydrogen bonds between the platinum amines and adjacent bases that were not evident from the more static NMR and crystal structures (Table 1). The hydrogen bond data are of intrinsic interest because they provide insight into a potential mechanistic explanation of differences in the solvent accessibility on the 5′ side of Pt-AGG and Pt-TGG adducts that had been suggested by the NMR data showing faster water exchange for imino protons when T is on the 5′ side of the Pt-GG adduct than when A is on the 5′ side of the adduct (Figure 5 and [46], [58]). Our MD data show that the Pt-amines frequently form hydrogen bonds with the 5′ A of Pt-AGG adducts, but not with the 5′ T of Pt-TGG adducts. (current work and [52]). Since the formation of a hydrogen bond with the 5′ A would be expected to restrict the conformational flexibility of the A•T base pair of the Pt-AGG adducts, the MD data provide a mechanistic explanation for the differences in solvent accessibility on the 5′ side of Pt-AGG and Pt-TGG adducts that had been suggested by the NMR data.

Our previous studies [52], [53] have also shown that identification of unique hydrogen bond patterns for CP-GG and OX-GG adducts in different sequence contexts can shed considerable insight into the specificity of protein-Pt-DNA binding because each hydrogen bond pattern is associated with a unique DNA conformation and some of these conformations may be particularly favorable templates for HMGB1a binding. For example, HMGB1a exhibits a large preference for binding CP-DNA in the TGGA sequence context and a slight preference for binding CP-DNA in the AGGC sequence context, while there is little or no difference in binding affinity of HMGB1a to CP- and OX-DNA in the TGGT sequence context [7], [10], [28], [59]. Our data show that in the TGGA sequence context the formation of a hydrogen bond between the 3′ amine and A8-N7 was associated with a conformational distribution favorable for HMGB1a binding, and this hydrogen bond could only be formed by CP-GG adducts [53]. In the AGGC sequence context, the formation of a hydrogen bond between the 5′ Pt-amine and the A5-N7 was associated with a conformational distribution favorable for HMGB1a binding, and this hydrogen bond formed slightly more frequently for CP-GG adducts than for OX-GG adducts [52]. In the TGGT sequence context, CP-GG adducts form hydrogen bonds between the 3′ amine and T8-O4 much more frequently than OX-GG adducts, but the conformations associated with the formation of this hydrogen bond offer little or no obvious binding advantage for HMGB1a (Figures 8 and 9). Thus, our simulations provide consistent explanations for the differential binding affinity of HMGB1a to CP- and OX-DNA in three different sequence contexts.

In summary, we have used both NMR and MD simulations to characterize the average conformation and conformational dynamics of the OX-TGGT adduct. We have compared these structures to the binding specificity of HMGB1a for Pt-GG adducts because the structure of the HMGB1a-CP-GG DNA complex is known [40] and the binding specificity of HMGB1a to Pt-GG adducts is particularly well characterized [7], [10], [28], [59]. A strength of our work is that the methods used to obtain the solution structures of CP-GG adducts, OX-GG adducts and undamaged DNA in the AGGC sequence context [46], [58] and OX-GG adducts in the TGGT sequence context were identical. Similarly, our MD simulations of CP-, OX-DNA and undamaged DNA in three different sequence contexts have been performed using the same Pt parameters and computational approach [52], [53]. The inclusion of undamaged DNA in our studies was important because it allowed us to exclude conformational differences that were primarily due to the effect of sequence context on undamaged DNA conformation (Figure S4). Our NMR data provide a structural explanation for the preferential binding of HMGB1a to Pt-GG adducts in the TGGT sequence context compared to the AGGC sequence context. Our MD simulations allowed us to identify hydrogen bonds between the Pt-amines and adjacent bases that form only transiently and the low abundance DNA conformations associated with specific H-bond formation. This information provides a structural explanation for the effect of sequence context on the relative affinity of HMGB1a for CP-GG adducts and OX-GG adducts in three different sequences contexts.

## Materials and Methods

### Preparation, purification and characterization of the OX-TGGT Adduct

**Figure 10:**
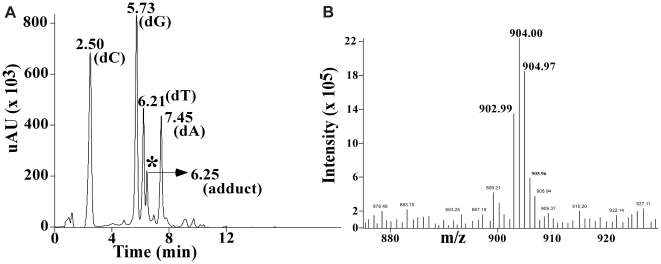
Purity of the duplex DNA used for NMR experiments. The spectra include the HPLC−UV elution profile of the digestion products obtained from the 12-mer duplex containing the OX−GG adduct [the peak with an asterisk corresponds to the elution position of a OX−d(GpG) standard] (**A**), and MS-positive ion mass spectrum of the peak identified with the asterisk in Figure 2A (**B**).

The 12-mer oligonucleotide containing the TGGT sequence (Figure 1) was platinated according to our previously described protocols and purified using HPLC (described in Text S1). Following hybridization with complementary strand, the OX-GG 12-mer duplex was further characterized following the same LC/MS procedure reported previously by Wu *et al*. [45], [46] Using this procedure, the Pt-GG-intrastrand cross-link is digested to Pt[d(GpG)] and Pt-G monoadducts to Pt(dG). In addition, both Pt-GNG-intrastrand cross-links and Pt-GG-interstrand cross-links are digested to dG-Pt-dG [60]. The digest of undamaged DNA showed peaks corresponding to the four normal deoxynucleosides (data not shown). The digest of the same 12-mer duplex containing the OX-DNA adduct showed the same four deoxynucleosides and one additional peak eluting just after dT (Figure 10A). This additional digestion product had the same retention time and UV spectrum as a synthetic OX[d(GpG)] standard (data not shown). This additional peak was further identified as OX[d(GpG)] by the presence of the expected molecular ions in both the positive (m/z 904.97) (Figure 10B) and negative (m/z 902.99) mass spectra. Lastly, the MS-spectra also showed the expected Pt-isotope pattern, confirming the presence of a Pt compound. No digestion products were detected with the masses and isotopic pattern expected for the dG-OX-dG or OX(dG) adducts. These data demonstrate that the 12-mer duplex employed in this NMR study consisted exclusively of the OX-GG-intrastrand cross-link, confirming the purity of the substrate used for structure determination.

### NMR Data Aquisition

For both undamaged DNA and the OX-DNA adduct in the TGGT sequence context, two NMR samples were prepared; one, H_2_O sample, in a 5% D_2_O/95% H_2_O buffer (100 mM NaCl, 5 mM phosphate buffer, pH 7.0) that was used for detection of exchangeable protons with varying temperature (2 to 40 °C) and the other, D_2_O sample, in 100% D_2_O buffer (same buffer composition as that of the H_2_O buffer) for detection of non-exchangeable protons. For both the samples, the duplex DNA concentration was 1.2 mM. NMR spectra were acquired on Varian Inova 500, 700, or 800 MHz spectrometers. The carrier frequency for protons was set on the H_2_O signal. 1D proton spectra were recorded using a Varian Inova 800 MHz NMR spectrometer at temperatures ranging from 2 to 40 °C for detection of exchangeable protons. NOESY spectra were recorded in D_2_O buffer at 25 °C at 700 MHz using a mixing time of 200 ms, 32 transients and 400 complex FIDs corresponding to spectral width of 25 and 12 ppm in both dimensions for both samples in H_2_O and D_2_O. To determine the optimal mixing time for quantification of NOE data, a series of 2D NOE data were collected in which the mixing times were varied (150 ms, 200 ms, 250 ms, and 300 ms). Close inspection of the data revealed that NOE data collected at a mixing time of 200 ms optimized signal/noise while minimizing spin diffusion effects. The WATERGATE pulse sequence was employed for water suppression in both H_2_O and D_2_O samples [61]. Distance constraints were obtained from the 200 ms NOESY spectra in both H_2_O and D_2_O [43]. The assignments were obtained initially from NOE connectivities and were confirmed by analysis of 2D DQF-COSY [44] (2048 × 720 complex points, 12 ppm spectral width in both dimensions, 32 transients). 2D DQF-COSY data were also used for determination of *J* coupling constraints for determination of sugar pucker as described previously [45], [46]. The J-coupling constants were estimated by simulating DQF-COSY cross-peaks using the program Chords 2.0 (Spectrum Research, LLC). All J_H2′–H2″_ values were set as −14.0 Hz. All other coupling constants were determined by adjusting their values in steps of 0.1 Hz. This step was repeated until the simulated multiplet looked very similar to its experimental counterpart. NMR data were processed with NMRPipe and analyzed with Felix (version 2000, Molecular Simulations, Inc., San Diego, CA). The structure calculations are essentially as reported previously [45], [46] and are described in detail in Text S1.

### Proton assignments

Chemical shift assignments for OX-DNA and undamaged DNA duplex in the TGGT sequence context were obtained essentially as described previously for the OX- and CP-DNA adduct in the AGGC sequence context [45], [46]. Assignment of the non-exchangeable base and sugar protons were obtained by analysis of NOESY and 2D DQF-COSY spectra as described in Methods. For example, the NOESY region for OX-DNA in Figures S1 and S2 shows NOE correlations between the base (purine H8/pyrimidine H6) and the H1′, H2′, and H2′′ sugar protons. Sequential connectivities can be observed without interruption from C1 to C12 in the GG strand and from G13 to G24 in the CC strand (Figures S1 and S2, panels A and B). Similar connectivities in the NOESY spectrum were also observed for the undamaged DNA in the same TGGT sequence context (Figures S1 and S2, panels C and D). Upon completion of the sequential assignments for the H8/H6, H1′, H2′, and H2′′ proton signals, assignments of other (H3′, H4′, H5′, and H5′′) sugar protons were obtained by following standard procedures with the NOESY and DQF-COSY spectra [62]. Assignment of the exchangeable protons was obtained by analyzing distance connectivities between the imino and base/amino proton regions of NOESY spectra in H_2_O buffer at 2°C. The chemical shift assignments for the OX-DNA duplex and undamaged DNA are shown in Tables S1 and S2, respectively.

### NMR Structure Determination

We obtained 481 and 665 experimental distance constraints for OX-DNA duplex and undamaged DNA respectively. These experimental distance constraints were used as input for CNS to calculate the respective solution structures of OX-DNA duplex and undamaged DNA as described in Methods. The structural statistics for OX-DNA duplex and undamaged DNA are listed in Table 2. Of 20 calculated structures for OX- and undamaged DNA, 14 with lowest energies were accepted as a family. The root mean square deviation (RMSD) for the superimposition of the heavy atoms for all 14 final structures was 0.83 Å for OX-DNA duplex and 1.21 Å for undamaged DNA.

**Table 2:** Structural statistics for both OX-DNA and undamaged-DNA structures in the TGGT sequence context.

Structure Related Information	OX-DNA	Undamaged DNA
**A. Experimental Distance Constraints**		
Intra-residue	315	432
Inter-residue	112	233
Cross-strand	54	118
Total	481	665
**B. Empirical Constraints**		
Hydrogen bond	72	72
Backbone Dihedral Angles	168	168
**C. Structural Statistics**		
Distance Violation per structure (>0.5 Å)	0	0
Dihedral Angle Violations per structure (>5°)	0	0
RMSD from ideal covalent geometry		
Bond Length (Å)	0.00 ± 0.01	0.01 ± 0.01
Bond Angle (°)	0.69 ± 0.01	0.46 ± 0.01
Dihedral Angle (°)	1.859 ± 0.01	0.00 ± 0.00
**D. Structure Quality**		
RMSD to the mean structure within the family (Å)		
All atoms	1.00 ± 0.30	1.16 ± 0.38
Non H atoms	0.83± 0.26	1.21 ± 0.30

### Starting Structures for Molecular Dynamics Simulations

We performed simulations on a 12-mer DNA sequence (similar to the one used for NMR experiments), which was either undamaged or covalently bound to CP or OX at the N7 of G6 and G7. There were two sets of simulations with different starting structures for each of CP-, OX- and undamaged DNA. The starting structures for CP- and OX-DNA were NMR and crystal structures of the adducts in the TGGT sequence context (1A84 (crystal) and 1AIO (NMR) for CP-DNA and 1IHH (crystal) and the average structure from this study (NMR) for OX-DNA). For undamaged DNA, the average NMR structure obtained in this study in the TGGT sequence context and the B-DNA structure in the TGGT sequence context (generated using Insight II) were used as the two starting structures.

### Molecular dynamics simulations

We performed 5 sets of 10 ns simulations for each starting structure of CP-, OX-, and undamaged DNA. The 5 sets had the same starting structures but the initial velocities were randomized. We employed simulation protocols identical to our published work on the AGGC [52] and TGGA sequence contexts [53].

### Analysis: Hydrogen Bonds

All the trajectories were analyzed for the presence of hydrogen bonds between all possible donors and acceptors based on a distance cut-off of 3.5 Å between the donor and acceptor and an angular cut-off of 135° between donor-H-acceptor. In addition to the Watson-Crick interactions the only hydrogen bonds that were observed for more than 10% of simulation time were between Pt-amines and the surrounding base pairs. All the frames of the trajectory were classified based on the type of hydrogen bonds formed between Pt-amines and the adjacent bases.

### Analysis: Helical parameters

Helical parameters were calculated for each snapshot of the trajectory using the CURVES program, version 5.3 [56], [57]. The analysis was performed using protocols identical to our earlier studies [52], [53]. The detailed methodology used for analysis is described in the Text S1.

## Supporting Information

Figure S1 **Expanded regions of a homonuclear 2D-NOESY (200 ms, D_2_O) spectrum showing** **H6/H8–H1′ sequential connectivities acquired on the 12-mer OX-TGGT sample and undamaged TGGT DNA duplex at 25°C and 700 MHz.** The regions containing H6/H8–H1′ sequential connectivities for the GG strand (**A** and **C**) and CC strand (**B** and **D**) are shown. (**A**) and (**B**) correspond to the OX-GG whereas (**C**) and (**D**) represent the undamaged GG duplex.
(TIF)

Figure S2 **Expanded regions of a homonuclear 2D-NOESY (200 ms, D_2_O) spectrum showing H6/H8–H2′/ H2″ sequential connectivities collected on 12-mer OX-GG and undamaged GG DNA duplex at 25°C and 700 MHz.** H6/H8–H2′/ H2′′ sequential connectivities for the GG strand (**A** and **C**) and CC strand (**B** and **D**) are shown. (**A**) and (**B**) correspond to the OX-TGGT duplex and (**C**) and (**D**) correspond to the undamaged TGGT duplex. Thick and dashed lines show H2′′ and H2′, respectively. (**) designate upfield-shifted H2′ resonances for T5, C18, and C19 in the 12-mer OX-GG duplex, compared to those in the undamaged 12-mer GG duplex (*).
(TIF)

Figure S3 **Root-mean-square deviation (RMSD) values for the MD simulations plotted as a function of time.** The RMSD values for each of the 5 simulations compared to the corresponding starting structure for the undamaged DNA, CP-DNA and OX-DNA are shown for the full 10 ns of each simulation. RMSD at time (t) represents the average of RMSD in a 250 ps bin centered at t (running average). The five simulation trajectories performed using the NMR structure of undamaged DNA, X-ray crystal structures and NMR structures of CP-DNA and OX-DNA as starting structures with different initial MD velocities are represented in black, red, blue, green and violet. The starting structure corresponding to each plot is represented as CP CRY, OX CRY, CP NMR, OX NMR and BDNA for crystal structure of CP-DNA, crystal structure of OX-DNA, NMR structure of CP-DNA, NMR structure of OX-DNA and the NMR structure of undamaged DNA in the TGGT sequence context.
(TIF)

Figure S4 **Helical parameters showing sequence specific effects while comparing OX-DNA and undamaged DNA in the TGGT and AGGC sequence context.** Histograms of the helical parameters showing the most significant differences between either OX-DNA or undamaged DNA in the TGGT and AGGC sequence contexts are plotted. The frequency distribution for a particular MD ensemble was obtained from the structures corresponding to the final 6 ns of each simulation, resulting in 60000 structures for undamaged DNA and 60000 structures for OX-DNA being used for histogram construction. The distributions of undamaged DNA and OX-DNA in the AGGC sequence context are plotted with solid and dashed black lines, respectively. The distributions of undamaged DNA and OX-DNA in the TGGT sequence contexts are plotted with solid and dashed red lines, respectively. The helical parameters shown are: 5–6 slide (**A**), 5–20 propellor twist (**B**), 7–18 buckle (**C**), 8–17 propellor twist (**D**), 5–20 buckle (**E**), 5–6 roll (**F**). The value of the each corresponding helical parameter in the crystal structure of HMGB1a-CP-DNA is indicated with a dashed vertical line.
(TIF)

Figure S5 **Conformational differences between different hydrogen bonded species in CP- and OX-DNA in the TGGT sequence context.** The conformational differences in the central four base pairs between structures forming G7-O6 hydrogen bond and structures forming no hydrogen bond to the drug; between structures forming T8-O4 hydrogen bond and structures forming no hydrogen bond to the drug and between structures forming the T8-O4 hydrogen bond and structures forming the G7-O6 hydrogen bond are plotted for CP-DNA (**A**) and OX-DNA (**B**) in the TGGT sequence context. The differences are represented as the KS ratio (described in Methods) displayed on a heat map. The heat map is color-coded and the KS ratio decreases in the order of Black to White according to the scale shown at the bottom of the heat map.
(TIF)

Text S1 **Supplemental NMR Experimental Procedures.**
(DOC)

Table S1 **1H NMR shifts (ppm) of the OX-DNA in the TGGT sequence context recorded in D2O buffer and at 25 °C.**
(DOC)

Table S2 **^1^H NMR shifts (ppm) of non platinated DNA in the TGGT sequence context recorded in D_2_O buffer at 25°C.**
(DOC)

Table S3 **Helical parameters of the NMR structures.**
(DOC)
