# Supplementary figures and images for: Flanking Bases Influence the Nature of DNA Distortion by Platinum 1,2-Intrastrand (GG) Cross-Links
Source: PLoS One. 2011 Aug 10;6(8):e23582. doi: 10.1371/journal.pone.0023582 (PMC3154474; doi:10.1371/journal.pone.0023582)

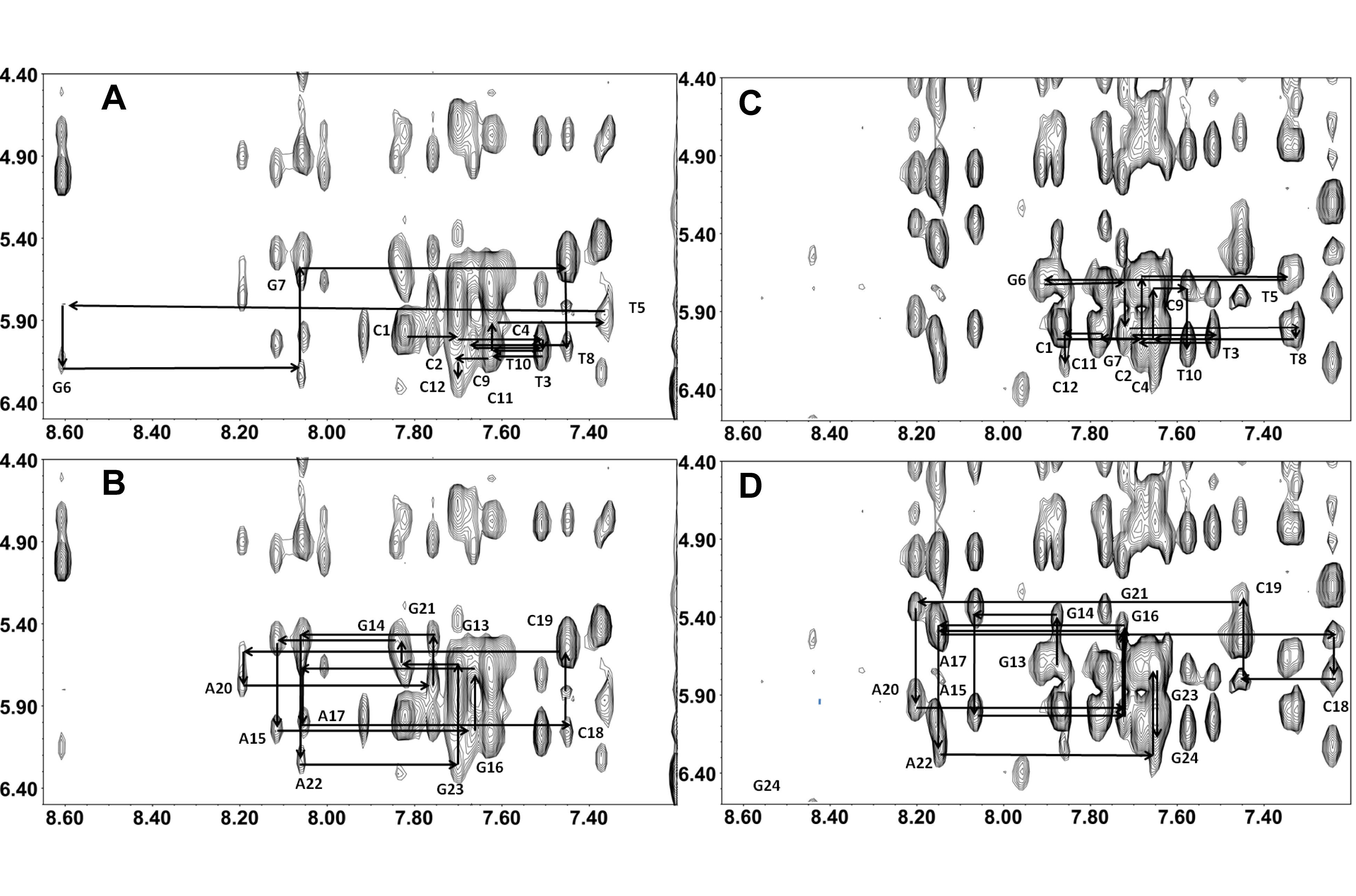

Supplement: Figure S1 — Expanded regions of a homonuclear 2D-NOESY (200 ms, D2O) spectrum showing H6/H8–H1′ sequential connectivities acquired on the 12-mer OX-TGGT sample and undamaged TGGT DNA duplex at 25°C and 700 MHz. The regions containing H6/H8–H1′ sequential connectivities for the GG strand (A and C) and CC strand (B and D) are shown. (A) and (B) correspond to the OX-GG whereas (C) and (D) represent the undamaged GG duplex. (TIF) [file pone.0023582.s001.tif]

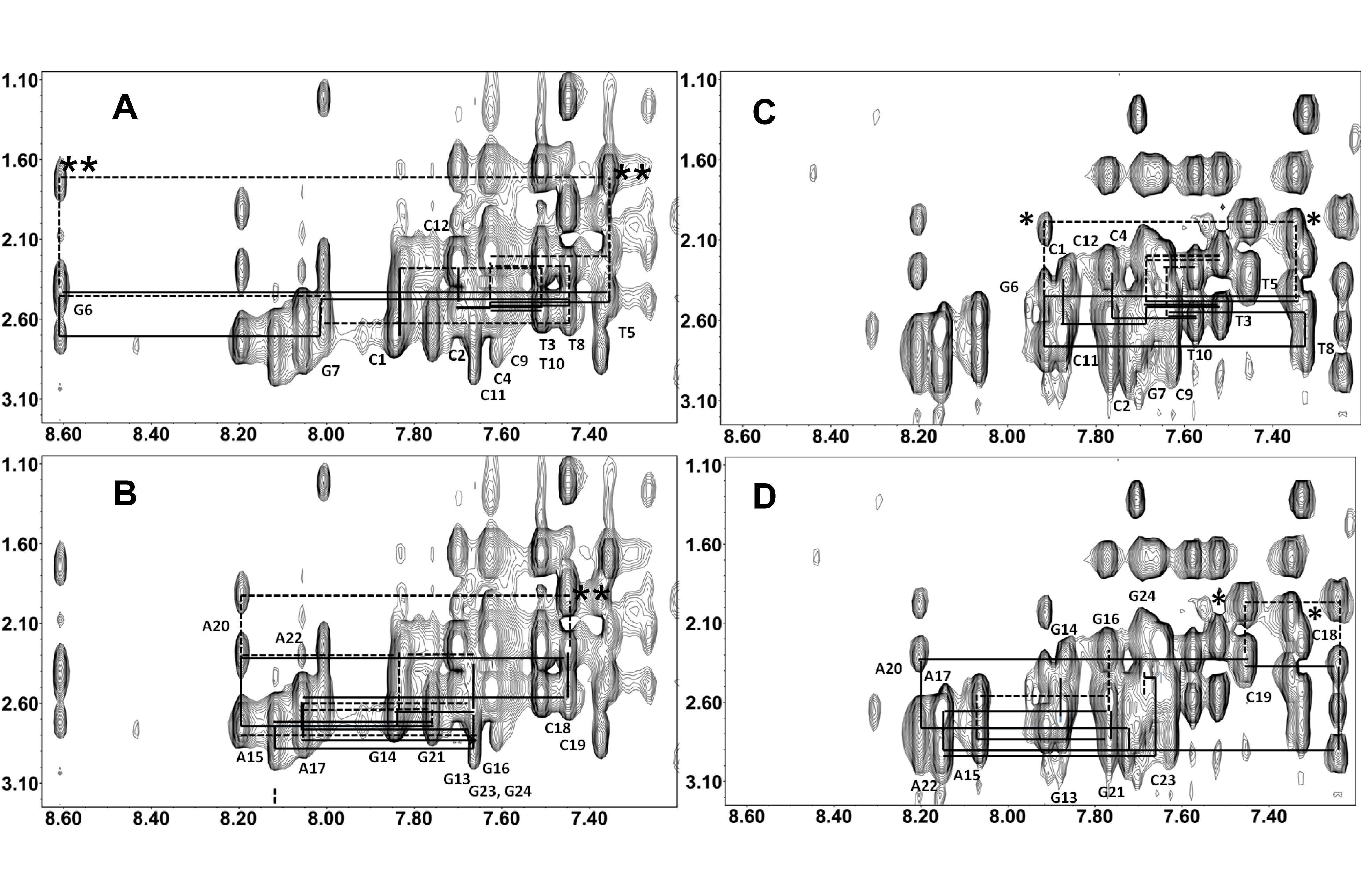

Supplement: Figure S2 — Expanded regions of a homonuclear 2D-NOESY (200 ms, D2O) spectrum showing H6/H8–H2′/ H2″ sequential connectivities collected on 12-mer OX-GG and undamaged GG DNA duplex at 25°C and 700 MHz. H6/H8–H2′/ H2′′ sequential connectivities for the GG strand (A and C) and CC strand (B and D) are shown. (A) and (B) correspond to the OX-TGGT duplex and (C) and (D) correspond to the undamaged TGGT duplex. Thick and dashed lines show H2′′ and H2′, respectively. (**) designate upfield-shifted H2′ resonances for T5, C18, and C19 in the 12-mer OX-GG duplex, compared to those in the undamaged 12-mer GG duplex (*). (TIF) [file pone.0023582.s002.tif]

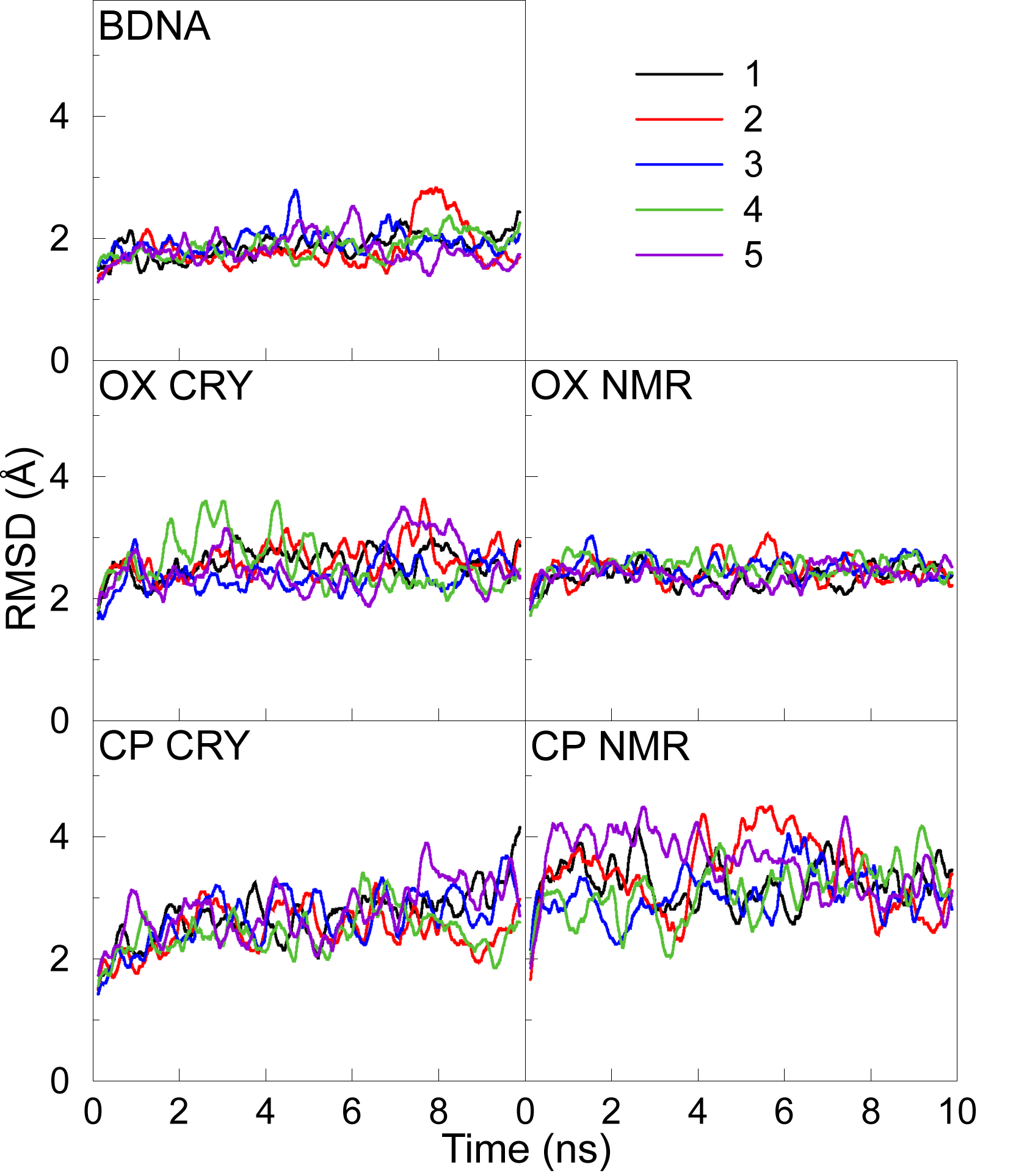

Supplement: Figure S3 — Root-mean-square deviation (RMSD) values for the MD simulations plotted as a function of time. The RMSD values for each of the 5 simulations compared to the corresponding starting structure for the undamaged DNA, CP-DNA and OX-DNA are shown for the full 10 ns of each simulation. RMSD at time (t) represents the average of RMSD in a 250 ps bin centered at t (running average). The five simulation trajectories performed using the NMR structure of undamaged DNA, X-ray crystal structures and NMR structures of CP-DNA and OX-DNA as starting structures with different initial MD velocities are represented in black, red, blue, green and violet. The starting structure corresponding to each plot is represented as CP CRY, OX CRY, CP NMR, OX NMR and BDNA for crystal structure of CP-DNA, crystal structure of OX-DNA, NMR structure of CP-DNA, NMR structure of OX-DNA and the NMR structure of undamaged DNA in the TGGT sequence context. (TIF) [file pone.0023582.s003.tif]

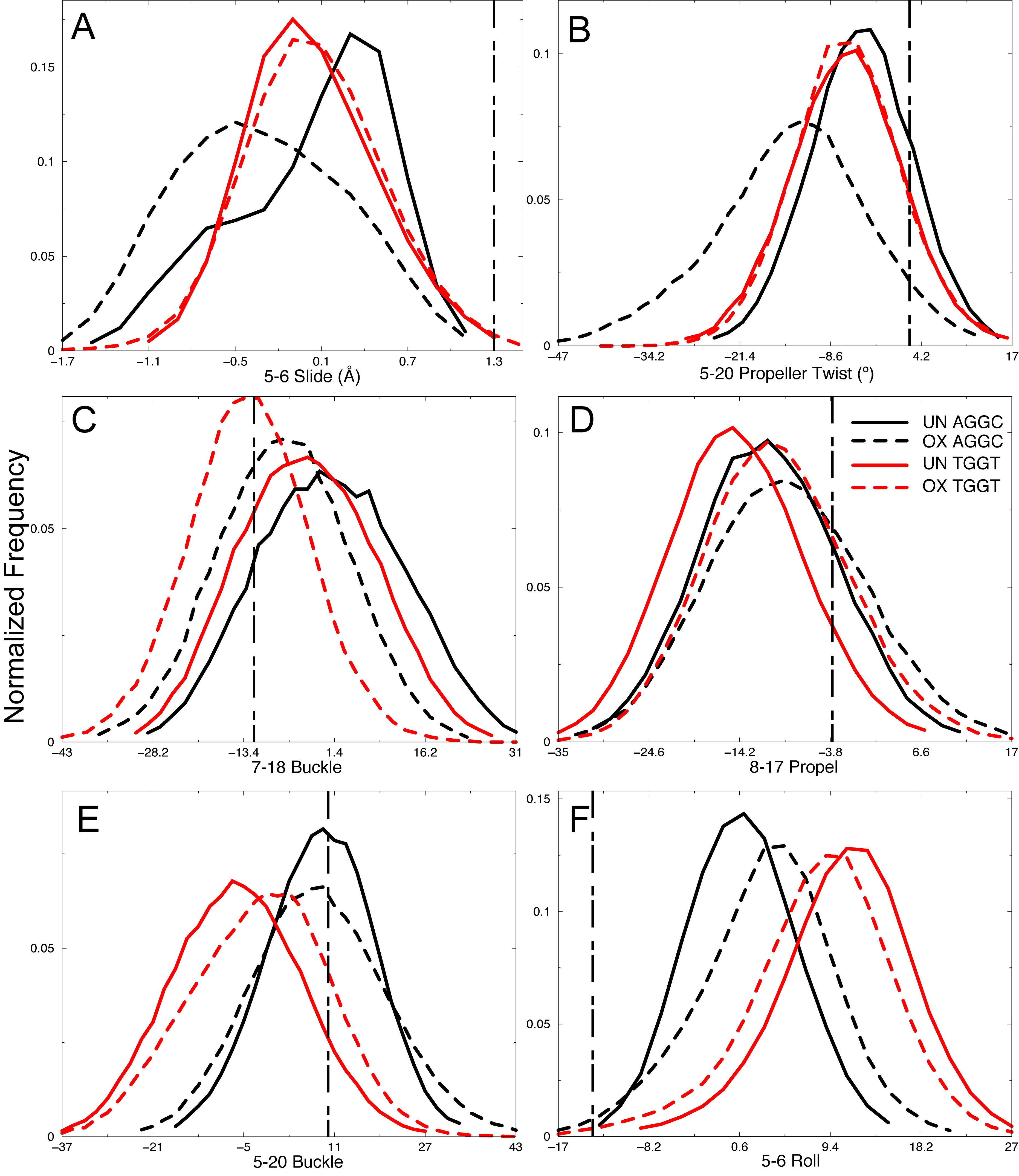

Supplement: Figure S4 — Helical parameters showing sequence specific effects while comparing OX-DNA and undamaged DNA in the TGGT and AGGC sequence context. Histograms of the helical parameters showing the most significant differences between either OX-DNA or undamaged DNA in the TGGT and AGGC sequence contexts are plotted. The frequency distribution for a particular MD ensemble was obtained from the structures corresponding to the final 6 ns of each simulation, resulting in 60000 structures for undamaged DNA and 60000 structures for OX-DNA being used for histogram construction. The distributions of undamaged DNA and OX-DNA in the AGGC sequence context are plotted with solid and dashed black lines, respectively. The distributions of undamaged DNA and OX-DNA in the TGGT sequence contexts are plotted with solid and dashed red lines, respectively. The helical parameters shown are: 5–6 slide (A), 5–20 propellor twist (B), 7–18 buckle (C), 8–17 propellor twist (D), 5–20 buckle (E), 5–6 roll (F). The value of the each corresponding helical parameter in the crystal structure of HMGB1a-CP-DNA is indicated with a dashed vertical line. (TIF) [file pone.0023582.s004.tif]

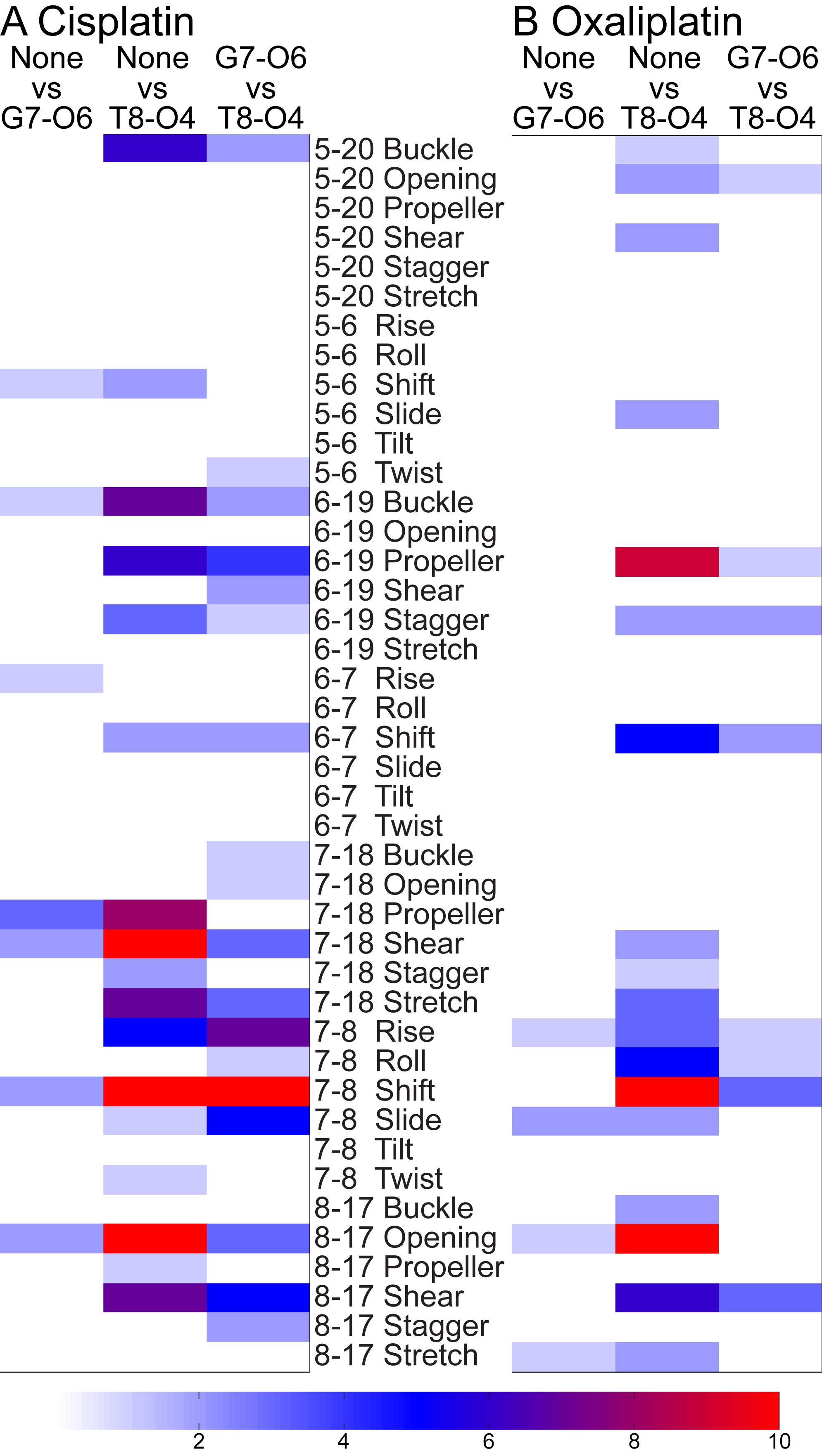

Supplement: Figure S5 — Conformational differences between different hydrogen bonded species in CP- and OX-DNA in the TGGT sequence context. The conformational differences in the central four base pairs between structures forming G7-O6 hydrogen bond and structures forming no hydrogen bond to the drug; between structures forming T8-O4 hydrogen bond and structures forming no hydrogen bond to the drug and between structures forming the T8-O4 hydrogen bond and structures forming the G7-O6 hydrogen bond are plotted for CP-DNA (A) and OX-DNA (B) in the TGGT sequence context. The differences are represented as the KS ratio (described in Methods) displayed on a heat map. The heat map is color-coded and the KS ratio decreases in the order of Black to White according to the scale shown at the bottom of the heat map. (TIF) [file pone.0023582.s005.tif]
