# Supplementary material for: Flanking Bases Influence the Nature of DNA Distortion by Platinum 1,2-Intrastrand (GG) Cross-Links
Source: PLoS One. 2011 Aug 10;6(8):e23582. doi: 10.1371/journal.pone.0023582 (PMC3154474; doi:10.1371/journal.pone.0023582)
Supplement: Text S1 — Supplemental NMR Experimental Procedures. (DOC) [file pone.0023582.s006.doc]

Flanking Bases Influence the Nature of DNA Distortion by Platinum 1,2-Intrastrand (GG) Cross-links

Bhattacharyya *et al.*

**Text** **S1**

**Supplemental NMR Experimental Procedures.**

**Preparation and HPLC Purification of the OX-TGGT Adduct.** The 12-mer containing the TGGT sequence and the complementary 12-mer were purchased from Operon Biotechnologies, Inc, Alabama, USA. The TGGT strand containing the oxaliplatin adduct was prepared using our previously described procedure [1] with slight modifications [2]. The platinated 12-mer TGGT strand was separated from the non-platinated TGGT strand by HPLC using a Phenosphere 5μ SAX 80A column (Phenomenex, Torrance, CA) with a 10 minute (20% – 68%) and 30 minute (68% - 78%) (w/v) gradient of buffer A (water) to buffer B (1 M NaCl) at a flow rate of 1 mL/min. The platinated-TGGT strand was dialyzed in water immediately after purification by HPLC to prevent loss of oxaliplatin-DNA adducts. Following desalting and concentration, the purity of the OX-adducted 12-mer TGGT strand was confirmed by denaturing gel electrophoresis. The DNA duplex (Figure 1) was prepared by adding the complementary strand solution to the TGGT strand in NMR buffer (100 mM NaCl, 5 mM phosphate buffer, pH 7.0) at a calculated 1:1 ratio. The DNA was annealed by cooling overnight from 70 ºC to room temperature. The purity of DNA duplex was confirmed by monitoring T-CH_3_ signal shifts and intensities, and signal intensities of the C1- and C18-H6 signals by both 1D ^1^H NMR and 2D DQF-COSY NMR spectra. Results from these analyses indicated a 1:1 ratio of TGGT strand to complementary strand.

**Characterization of the OX-TGGT Adduct by LC/MS.** Following hybridization with complementary strand, the OX-TGGT 12-mer duplex was further characterized following the same LC/MS procedure reported previously by Wu *et al*. [1,2]. The OX[d(GpG)] adduct and undamaged DNA duplex in the same TGGT sequence contexts were digested with deoxyribonuclease I, nuclease P_1_, and alkaline phosphatase. The digested samples were analyzed on a Surveyor LC system, connected to Surveyor photo diode array (PDA) and LCQDECA ion trap mass spectrometer (Therm San Jose, CA). A Clarity 3μ Oligo-RP column (100 × 4.60 mm) was employed using a linear gradient of 100% 10 mM ammonium acetate (pH 3.5) to 80% MeOH in 10 mM ammonium acetate for 20 minutes at a flow rate of 200 μl/min. UV spectra (λ range of 200-400 nm) were recorded with a Finnigan Surveyor PDA detector, and positive and negative full scan (m/z 80-1000) mass spectra were acquired with Xcalibur.

**Structure calculations.** NMR solution structures of the DNA duplexes were calculated using the program CNS (Crystallographic and NMR System) version 1.1 employing a simulated annealing protocol, as described previously [1,2]. The bonds, angles, dihedral angles and force constants for the OX moiety were obtained from Scheef *et al*. [3] An initial extended structure was produced from the duplex sequence. Two types of constraints were used in the structure calculations: experimentally-derived and halonomic distance constraints. A total of 481 and 665 NOE-derived distance restraints were employed in the structure calculations for OX-GG DNA and undamaged DNA duplexes, respectively (Table S1). The NOE cross-peak intensities were standardized to the C H5-H6 cross-peaks, with a H5-H6 distance of 2.5 Å. One additional angstrom was added for distance constraints involving methyl groups. Planarity was also restrained for all base pairs [4] with the exception of the base pairs associated with the OX adduct, where the force constant was reduced by half. Although the sugar pucker conformation was estimated from analysis of NOE and DQF-COSY constraints, a broad range of torsion angle restraints were employed for the sugar-phosphate torsion angles in the structure calculations, i.e. α (-70 ± 50°), β (180 ± 50°), γ (60 ± 35°), ζ (-85 ± 50°),χ(-120 ± 50°), δ (120 ± 50°) and ε(180 ± 50°). Finally, six distance restraints per base pair were employed to describe Watson-Crick base pairing [4]. The lower and upper bounds for H-bond distance constraints were set to 7%. However, for base pairs associated with the OX adduct, the bounds were increased to 10%.

Among the 20 calculated structures for each OX-DNA adduct or undamaged DNA duplex, the 14 calculated structures with the lowest energy were accepted as a family (Supplemental Figure S5). PDB ID codes for the 14 lowest energy structures are 2k0t and 2k0v for the OX-TGGT DNA adduct and undamaged DNA, respectively. The RMSD deviation for the superimposition of the non-H atoms for all 14 final structures was 0.83 Å for OX-DNA and 1.21 Å for undamaged DNA in the TGGT sequence context, indicating good convergence for both structures. The fact that there were few or no distance or dihedral angle restraint violations > 0.5 Å and 5°, respectively, also indicated that the final structures satisfied the imposed experimental restraints.

**Conformational Analysis of the NMR Structures.** Helical parameters were calculated for each of the 14 lowest energy structures of OX-DNA and undamaged DNA in the TGGT and AGGC sequence contexts using the CURVES program, version 5.3 [5,6]. The helical parameters corresponding to the central four base-pairs were compared between different NMR structure ensembles using the Z-scores. The Z-score of comparison of 2 NMR ensembles is defined as:

$Zscore=\frac{(Mean_{1}-Mean_{2})}{(SD_{1}+SD_{2})}$

where Mean_1_ and SD_1_ are mean and the standard deviations of one of the NMR ensembles and Mean_2_ and SD_2_ are the same for the other NMR ensemble. The Z-scores of different comparisons were then represented as colors in a diagram (heat map) shown in Figure 4. The Z-scores transition from white (lowest Z-scores) to red (highest Z-scores) in the heat map.

**MD Experimental Procedures: Calculation of centroid structures.** We calculated the average structures for both the complete ensembles of CP-, OX- and undamaged DNA. Since average structures are not actual structures from simulations and may feature some abnormal bond lengths/angles, we assigned the structure from each ensemble that had the lowest root-mean-square deviation (RMSD) to the average structure as the centroid structure of that ensemble.

**Comparisons of centroid structures using RMSD.** We compared the centroid structures of CP- and OX-DNA adducts in TGGA and AGGC sequence contexts respectively. In these comparisons, we used only the atoms from the DNA part of the molecule in calculating the mass-weighted RMSD.

**Helical Parameters.** Histograms were constructed for each helical parameter corresponding to the central four base-pairs as frequency of occurrence versus discrete units of each DNA helical parameter. The discrete units (bin width) were set as 0.2 Å for all the distance parameters and 2º for all the angular parameters. These distributions were also clustered on the basis of the pattern of hydrogen bond formation between the DNA and the drug.

To calculate the *P*-value of the difference between helical parameters corresponding to CP-, OX-DNA and undamaged DNA and also between different hydrogen bonded species in CP- and OX-DNA, we used the Kolmogorov-Smirnov (KS) test. The lower cut-off threshold of significant differences was calculated by dividing each data set into 5 equal subsets and performing the KS test comparing all possible combinations of the 5 subsets of divided data. The highest –log(*P*) among the subsets of each data set was set as the threshold for that data set. When two data sets were compared, the difference between them was significant only if –log(*P*) of the comparison of the data sets was higher than the threshold. The ratio of –log(*P*) of the comparison of the data sets to their thresholds was calculated (KS-ratio) and then this ratio for all the parameters are represented as colors in a diagram (heat map). The ratios transition from white (lowest ratios) to black (highest ratios) in the heat map.

**Comparison of MD and NOE distances.** To ensure that the ensemble obtained through the MD simulations was representative of the experimental NMR data, we determined the distances between pairs of atoms whose NOE constraints are known in the central four base-pairs. In the CNS calculations (for NMR structure determination), a distance violation is defined as an inter-proton distance deviating from the range of corresponding distance constraints by more than 0.5 Å. The same criterion was applied here when we tried to evaluate how well our MD simulation structures agreed with the NMR data using the NMR distance constraints obtained for the central four base pairs 5′-d(T5G6G7T8)-3 (Table S4, shown below). We observed only six out of the 178 restraints determined in the central four base-pairs to be violated in the OX-DNA simulations (Table S4). Of the six violations, five involved sugars and those violations could be a result of systematic preference of the AMBER DNA force field for certain sugar puckers. This preference was further substantiated by analysis of the NOE violations from the simulations of undamaged DNA, where we observe a number of NOE constraints involving sugar hydrogens being violated (Table S4). Thus, the MD ensembles are in good agreement with the NMR NOE restraints with the possible exception of those involving sugar pucker.

**Supplemental Table S4. Summary of NOE violations for OX-DNA.**

| Type | Pair | NMR Mean (Å) | NMR SD (Å) | MD Mean (Å) | MD SD (Å) | NOE-score (Å) | Z-score |
| --- | --- | --- | --- | --- | --- | --- | --- |
| OX-DNA | | | | | | | |
| Base-Sugar | 6 H8 - 6 H3' | 3.00 | 0.75 | 4.26 | 0.81 | 0.01 | 0.81 |
| Base-Sugar | 20 H8 - 20 H5'' | 3.40 | 0.85 | 5.40 | 0.58 | 0.66 | 1.40 |
| Base-Sugar | 7 H8 - 7 H5' | 2.56 | 0.64 | 4.77 | 0.30 | 1.07 | 2.34 |
| Base-Sugar | 7 H8 - 6 H3' | 3.66 | 0.92 | 5.42 | 0.87 | 0.34 | 0.98 |
| Base-Sugar | 8 H6 - 8 H5' | 2.85 | 0.71 | 4.34 | 0.42 | 0.27 | 1.31 |
| Base-Base | 6 H1 - 7 H22 | 7.67 | 1.92 | 5.23 | 0.57 | 0.02 | 0.98 |
| Undamaged DNA | | | | | | | |
| Intra Base | 5 H3 - 5 H7# | 3.10 | 0.78 | 4.94 | 0.07 | 0.56 | 2.16 |
| Base-Base | 8 H3 - 18 H41 | 3.74 | 0.94 | 5.90 | 0.53 | 0.72 | 1.47 |
| Intra Base | 8 H3 - 8 H7# | 3.29 | 0.82 | 4.93 | 0.07 | 0.32 | 1.83 |
| Base-Sugar | 6 H8 - 6 H5' | 3.34 | 0.83 | 5.06 | 0.59 | 0.39 | 1.21 |
| Base-Sugar | 6 H8 - 6 H3' | 3.20 | 0.80 | 4.59 | 0.24 | 0.08 | 1.32 |
| Base-Sugar | 7 H8 - 7 H3' | 2.91 | 0.73 | 4.44 | 0.39 | 0.30 | 1.37 |
| Base-Sugar | 5 H6 - 5 H5' | 3.01 | 0.75 | 4.54 | 0.46 | 0.28 | 1.26 |
| Base-Sugar | 8 H6 - 7 H1' | 2.79 | 0.70 | 4.16 | 0.66 | 0.17 | 1.01 |
| Base-Sugar | 7 H1' - 8 H7# | 3.13 | 0.78 | 5.35 | 0.48 | 0.94 | 1.77 |
| Sugar-Sugar | 7 H1' - 8 H5' | 1.86 | 0.46 | 3.24 | 0.74 | 0.42 | 1.15 |
| Sugar-Sugar | 18 H1' - 19 H5' | 1.93 | 0.48 | 3.23 | 0.64 | 0.32 | 1.16 |
| Sugar-Sugar | 6 H1' - 7 H5' | 1.91 | 0.48 | 3.71 | 1.01 | 0.82 | 1.21 |
| Base-Sugar | 19 H5 - 19 H2'' | 4.14 | 1.03 | 5.72 | 0.30 | 0.05 | 1.19 |
| Base-Sugar | 18 H5 - 18 H5' | 4.77 | 1.19 | 6.59 | 0.47 | 0.12 | 1.09 |
| Sugar-Sugar | 19 H1' - 20 H1' | 3.88 | 0.97 | 5.68 | 0.53 | 0.33 | 1.20 |
| Sugar-Sugar | 19 H1' - 20 H5' | 1.87 | 0.47 | 3.62 | 0.58 | 0.78 | 1.68 |
| Sugar-Sugar | 19 H1' - 20 H5'' | 3.34 | 0.83 | 5.25 | 0.55 | 0.58 | 1.38 |
| ^1^NOE-score denotes the extent of violation of the NOE constraint and is computed as  |MD_Mean_-  (NMR_Mean_+NMR_SD_ +0.5)|  ^2^Z-score is computed as |( NMR_Mean_­­- MD_Mean_)/( NMR_SD_+ MD_SD_)|; Z-scores > 1 indicate significant differences. | | | | | | | |

**Supplemental References.**

1. Wu Y, Pradhan P, Havener J, Boysen G, Swenberg JA, et al. (2004) NMR solution structure of an oxaliplatin 1,2-d(GG) intrastrand cross-link in a DNA dodecamer duplex. J Mol Biol 341: 1251-1269.

2. Wu Y, Bhattacharyya D, King CL, Baskerville-Abraham I, Huh SH, et al. (2007) Solution structures of a DNA dodecamer duplex with and without a cisplatin 1,2-d(GG) intrastrand cross-link: Comparison with the same DNA duplex containing an oxaliplatin 1,2-d(GG) intrastrand cross-link. Biochemistry 46: 6477-6487.

3. Scheeff ED, Briggs JM, Howell SB (1999) Molecular modeling of the intrastrand guanine-guanine DNA adducts produced by cisplatin and oxaliplatin. Molecular Pharmacology 56: 633-643.

4. Kuszewski J, Schwieters C, Clore GM (2001) Improving the accuracy of NMR structures of DNA by means of a database potential of mean force describing base-base positional interactions. JAmChemSoc 123: 3903-3918.

5. Lavery R, Skelnar H (1989) Defining the structure of irregular nucleic acids: Conventions and principles. Journal of Biomolecular Structure and Dynamics 6: 655-667.

6. Lavery R, Sklenar H (1988) The definition of generalized helicoidal parameters and of axis curvature for irregular nucleic acids. Journal of Biomolecular Structure and Dynamics 6: 63-91.
