# Supplementary material for: Flanking Bases Influence the Nature of DNA Distortion by Platinum 1,2-Intrastrand (GG) Cross-Links
Source: PLoS One. 2011 Aug 10;6(8):e23582. doi: 10.1371/journal.pone.0023582 (PMC3154474; doi:10.1371/journal.pone.0023582)
Supplement: Table S1 — 1H NMR shifts (ppm) of the OX-DNA in the TGGT sequence context recorded in D2O buffer and at 25 °C. (DOC) [file pone.0023582.s007.doc]

**Supplemental** **Table S1.** ^1^H NMR shifts (ppm) of the OX-DNA in the TGGT sequence context recorded in D_2_O buffer and at 25 °C

| **Residue** | **H6/**  **H8** | **H5/**  **Me/H2** | **H1′** | **H2′** | **H2′′** | **H3′** | **H4′** | **H5′** | **H5′′** | **NH^*^** | **NH_2_^*^** |
| --- | --- | --- | --- | --- | --- | --- | --- | --- | --- | --- | --- |
| C1 | 7.82 | 5.96 | 6.01 | 2.27 | 2.69 | 4.77 | 4.16 | 3.78 | 3.78 |  | 7.87/7.32 |
| C2 | 7.70 | 5.70 | 6.02 | 2.24 | 2.53 | 4.71 | 4.13 | 4.10 | 4.10 |  | 8.77/7.13 |
| T3 | 7.51 | 1.63 | 6.09 | 2.27 | 2.56 | 4.84 | 4.52 | 4.16 | 4.16 | 14.02 |  |
| C4 | 7.63 | 5.64 | 5.99 | 2.20 | 2.53 | 4.80 | 4.16 | 4.13 | 4.13 |  | 7.87/7.32 |
| T5 | 7.36 | 1.69 | 5.83 | 1.72 | 2.49 | 4.80 | 4.10 | 4.10 | 4.10 | 14.47 |  |
| G6 | 8.61 |  | 6.12 | 2.44 | 2.71 | 5.03 | 4.23 | 4.16 | 4.16 | 13.37 | 8.10/5.86 |
| G7 | 8.02 |  | 5.67 | 2.24 | 2.49 | 4.77 | 4.20 | 4.07 | 4.07 | 13.18 | 9.10/6.31 |
| T8 | 7.45 | 1.24 | 6.06 | 2.30 | 2.53 | 4.77 | 4.23 | 4.10 | 4.10 | 14.23 |  |
| C9 | 7.62 | 5.70 | 6.06 | 2.13 | 2.49 | 4.77 | 4.20 | 4.16 | 4.16 |  | 8.66/7.20 |
| T10 | 7.51 | 1.66 | 6.04 | 2.24 | 2.53 | 4.80 | 4.23 | 4.13 | 4.13 | 14.09 |  |
| C11 | 7.62 | 5.77 | 6.06 | 2.28 | 2.49 | 4.84 | 4.48 | 3.87 | 3.87 |  | 8.78/7.27 |
| C12 | 7.69 | 5.83 | 6.25 | 2.33 | 2.30 | 4.6 | 4.10 | 4.29 | 4.07 |  | 8.21/6.98 |
| G13 | 7.66 |  | 5.68 | 2.30 | 2.62 | 4.93 | 4.10 | 3.58 | 3.58 | 13.01 |  |
| G14 | 7.85 |  | 5.54 | 2.43 | 2.77 | 4.90 | 4.13 | 4.10 | 4.10 | 13.02 | 7.95/6.09 |
| A15 | 8.12 | 7.74 | 6.06 | 2.69 | 2.90 | 4.97 | 4.42 | 4.16 | 4.10 |  | 7.99/6.31 |
| G16 | 7.66 |  | 5.74 | 2.69 | 2.74 | 4.90 | 4.29 | 4.23 | 4.10 | 12.95 | 8.21/5.86 |
| A17 | 8.06 | 7.96 | 6.06 | 2.56 | 2.82 | 4.90 | 4.64 | 4.32 | 4.26 |  | 7.76/6.31 |
| C18 | 7.44 | 5.40 | 5.92 | 1.95 | 2.27 | 4.84 | 4.10 | 4.10 | 4.10 |  | 8.60/5.98 |
| C19 | 7.45 | 5.52 | 5.57 | 1.88 | 2.30 | 4.77 | 4.07 | 3.94 | 3.94 |  | 8.66/6.87 |
| A20 | 8.19 | 7.67 | 5.80 | 2.46 | 2.72 | 4.93 | 4.32 | 4.07 | 3.91 |  | 7.87/5.98 |
| G21 | 7.76 |  | 5.48 | 2.62 | 2.75 | 4.97 | 4.26 | 4.13 | 4.13 | 12.90 | 8.66/6.85 |
| A22 | 8.05 | 7.37 | 6.22 | 2.56 | 2.83 | 4.87 | 4.48 | 4.13 | 4.13 |  | 8.10/6.20 |
| G23 | 7.66 |  | 5.62 | 2.20 | 2.65 | 4.10 | 4.39 | 4.26 | 4.26 | 13.12 | 8.32/7.32 |
| G24 | 7.70 |  | 6.16 | 2.46 | 2.40 | 4.64 | 4.16 | 4.16 | 4.16 | 13.37 | 8.54 |

* - data recorded at 2 °C
