# Supplementary material for: Flanking Bases Influence the Nature of DNA Distortion by Platinum 1,2-Intrastrand (GG) Cross-Links
Source: PLoS One. 2011 Aug 10;6(8):e23582. doi: 10.1371/journal.pone.0023582 (PMC3154474; doi:10.1371/journal.pone.0023582)
Supplement: Table S2 — 1H NMR shifts (ppm) of non platinated DNA in the TGGT sequence context recorded in D2O buffer at 25°C. (DOC) [file pone.0023582.s008.doc]

**Supplemental Table S2**. ^1^H NMR shifts (ppm) of non platinated DNA in the TGGT sequence context recorded in D_2_O buffer at 25 °C.

| **Residue** | **H6/H8** | **H5/Me/H2** | **H1′** | **H2′** | **H2′′** | **H3′** | **H4′** | **H5′** | **H5′′** | **NH*** | **NH_2_^*^** |
| --- | --- | --- | --- | --- | --- | --- | --- | --- | --- | --- | --- |
| C1 | 7.86 | 5.20 | 6.04 | 2.37 | 2.62 | 4.78 | 4.16 | 3.84 | 3.26 |  | 7.99/5.41 |
| C2 | 7.69 | 5.79 | 6.03 | 2.18 | 2.56 | 4.84 | 4.36 | 4.20 | 4.20 |  | 8.51/7.09 |
| T3 | 7.51 | 1.70 | 6.09 | 2.23 | 2.51 | 4.94 | 4.49 | 4.20 | 3.53 | 13.80 |  |
| C4 | 7.68 | 5.71 | 6.07 | 2.21 | 2.46 | 4.84 | 4.23 | 4.20 | 4.20 |  | 7.42/6.33 |
| T5 | 7.34 | 1.73 | 5.70 | 2.03 | 2.46 | 4.87 | 4.16 | 3.75 | 3.75 | 13.86 |  |
| G6 | 7.90 |  | 5.74 | 2.32 | 2.75 | 4.84 | 4.42 | 4.16 | 4.16 | 12.73 | 7.18/5.75 |
| G7 | 7.69 |  | 5.97 | 2.56 | 2.80 | 4.86 | 4.45 | 4.21 | 4.16 | 12.70 | 7.34/5.24 |
| T8 | 7.32 | 1.34 | 6.10 | 2.24 | 2.53 | 4.84 | 4.35 | 4.21 |  | 13.61 |  |
| C9 | 7.64 | 5.67 | 5.84 | 2.27 | 2.56 | 4.81 | 4.16 | 3.68 | 3.68 |  | 8.34/7.09 |
| T10 | 7.57 | 1.73 | 6.06 | 2.24 | 2.59 | 4.87 | 4.44 | 4.20 | 4.20 | 13.74 |  |
| C11 | 7.76 | 5.76 | 6.03 | 2.30 | 2.59 | 4.78 | 4.42 | 4.20 | 4.18 |  | 8.34/7.42 |
| C12 | 7.85 | 5.60 | 6.26 | 2.34 | 2.30 | 4.73 | 4.40 | 3.73 | 3.23 |  | 8.76/ |
| G13 | 7.88 |  | 5.69 | 2.46 | 2.69 | 4.81 | 4.49 | 4.15 | 3.75 | 13.21 | 7.18/5.75 |
| G14 | 7.87 |  | 5.45 | 2.50 | 2.72 | 5.00 | 4.39 | 4.16 | 3.72 | 12.95 | 8.34/5.58 |
| A15 | 8.06 | 7.85 | 6.05 | 2.59 | 2.82 | 5.02 | 4.45 | 4.20 |  |  | 7.67/5.75 |
| G16 | 7.72 |  | 5.35 | 2.24 | 2.64 | 5.01 | 4.40 | 4.21 | 4.17 | 12.59 | 7.76/5.50 |
| A17 | 8.15 | 7.79 | 5.48 | 2.69 | 2.91 | 5.06 | 4.49 | 4.23 | 4.18 |  | 7.60/5.66 |
| C18 | 7.23 | 5.24 | 5.80 | 1.95 | 2.37 | 4.77 |  | 4.20 | 4.10 |  | 8.42/6.75 |
| C19 | 7.45 | 5.56 | 5.33 | 2.01 | 2.34 | 4.74 |  | 4.17 | 4.10 |  | 7.91/6.42 |
| A20 | 8.20 | 7.42 | 5.96 | 2.33 | 2.75 | 5.03 | 4.42 | 4.13 | 4.13 |  | 7.84/6.25 |
| G21 | 7.73 |  | 5.52 | 2.62 | 2.87 | 5.02 | 4.39 | 4.21 | 3.85 | 12.58 | 8.42/7.00 |
| A22 | 8.14 | 7.63 | 6.25 | 2.66 | 2.94 | 5.00 | 4.22 | 4.20 | 4.20 |  | 7.67/5.91 |
| G23 | 7.66 |  | 5.65 | 2.50 | 2.62 | 4.97 | 4.36 | 4.20 | 4.20 | 12.86 | 8.17/5.66 |
| G24 | 7.68 |  | 6.17 | 2.58 | 2.40 | 4.89 | 4.23 | 4.13 | 4.13 | 13.20 |  |

* - data recorded at 2 °C
