# Supplementary material for: Flanking Bases Influence the Nature of DNA Distortion by Platinum 1,2-Intrastrand (GG) Cross-Links
Source: PLoS One. 2011 Aug 10;6(8):e23582. doi: 10.1371/journal.pone.0023582 (PMC3154474; doi:10.1371/journal.pone.0023582)
Supplement: Table S3 — Helical parameters of the NMR structures. (DOC) [file pone.0023582.s009.doc]

**Supplemental Table S3. Helical parameters of the NMR structures.**

| **Helical Parameter** | **OX-TGGT** | **UN-TGGT** | **OX-AGGC** | **UN-AGGC** |
| --- | --- | --- | --- | --- |
| 5-20 Buckle | -1.38 ± 0.95 | -1.50 ± 0.56 | 2.41 ± 0.58 | 1.00 ± 1.58 |
| 5-20 Opening | -6.94 ± 1.55 | -0.72 ± 0.40 | 1.18 ± 0.98 | -2.37 ± 1.71 |
| 5-20 Propel | 1.58 ± 1.20 | -2.25 ± 0.85 | -4.35 ± 1.90 | -1.17 ± 2.13 |
| 5-20 Shear | -0.16 ± 0.13 | -0.07 ± 0.04 | -0.10 ± 0.04 | -0.39 ± 0.09 |
| 5-20 Stagger | 0.02 ± 0.02 | 0.00 ± 0.03 | -0.11 ± 0.02 | -0.09 ± 0.03 |
| 5-20 Stretch | -0.06 ± 0.09 | -0.20 ± 0.02 | -0.11 ± 0.06 | -0.26 ± 0.03 |
| 5-6 Rise | 3.73 ± 0.24 | 3.23 ± 0.25 | 3.12 ± 0.08 | 2.94 ± 0.07 |
| 5-6 Roll | -17.34 ± 6.41 | 1.51 ± 1.97 | -0.16 ± 0.72 | 3.11 ± 8.72 |
| 5-6 Shift | -0.58 ± 0.25 | -0.78 ± 0.18 | -0.06 ± 0.08 | 0.51 ± 0.13 |
| 5-6 Slide | 1.09 ± 0.19 | 0.73 ± 0.27 | -0.63 ± 0.07 | 0.17 ± 0.19 |
| 5-6 Tilt | -7.96 ± 1.46 | -5.27 ± 1.08 | -4.86 ± 0.66 | -2.91 ± 2.49 |
| 5-6 Twist | 36.77 ± 3.98 | 38.89 ± 1.67 | 23.72 ± 1.39 | 32.62 ± 2.89 |
| 6-19 Buckle | 6.19 ± 1.11 | 1.22 ± 1.43 | 12.60 ± 2.48 | 9.48 ± 2.00 |
| 6-19 Opening | 4.32 ± 0.54 | -0.31 ± 1.46 | 4.07 ± 0.29 | -0.29 ± 3.22 |
| 6-19 Propeller Twist | -4.90 ± 0.81 | -1.04 ± 0.86 | -11.10 ± 1.32 | 1.68 ± 3.04 |
| 6-19 Shear | -0.16 ± 0.15 | -0.07 ± 0.30 | -0.37 ± 0.07 | 0.13 ± 0.27 |
| 6-19 Stagger | -0.06 ± 0.04 | 0.05 ± 0.09 | -0.03 ± 0.02 | 0.23 ± 0.08 |
| 6-19 Stretch | -0.09 ± 0.05 | -0.09 ± 0.03 | -0.02 ± 0.03 | -0.15 ± 0.10 |
| 6-7 Rise | 4.05 ± 0.37 | 3.42 ± 0.13 | 3.09 ± 0.13 | 2.67 ± 0.26 |
| 6-7 Roll | 44.27 ± 3.98 | 4.44 ± 3.76 | 24.70 ± 3.15 | -3.17 ± 4.10 |
| 6-7 Shift | 0.42 ± 0.22 | 0.47 ± 0.12 | 0.81 ± 0.13 | 0.52 ± 0.20 |
| 6-7 Slide | -1.00 ± 0.33 | 0.10 ± 0.20 | -1.10 ± 0.19 | -1.99 ± 0.19 |
| 6-7 Tilt | -3.95 ± 1.44 | -3.59 ± 0.68 | -0.45 ± 0.42 | 0.92 ± 1.79 |
| 6-7 Twist | 25.40 ± 5.53 | 35.67 ± 2.31 | 26.99 ± 1.57 | 24.14 ± 2.78 |
| 7-18 Buckle | -4.31 ± 1.24 | 0.54 ± 0.80 | -2.24 ± 1.73 | 0.68 ± 1.53 |
| 7-18 Opening | 3.90 ± 1.34 | -1.07 ± 0.66 | 7.33 ± 0.52 | 1.98 ± 1.58 |
| 7-18 Propeller Twist | 6.74 ± 2.30 | 3.11 ± 2.21 | -0.32 ± 1.64 | -0.92 ± 3.72 |
| 7-18 Shear | -0.23 ± 0.09 | 0.56 ± 0.06 | -0.44 ± 0.16 | 0.32 ± 0.08 |
| 7-18 Stagger | -0.06 ± 0.04 | 0.06 ± 0.06 | 0.06 ± 0.03 | -0.11 ± 0.09 |
| 7-18 Stretch | -0.09 ± 0.07 | -0.02 ± 0.03 | 0.13 ± 0.09 | 0.01 ± 0.05 |
| 7-8 Rise | 3.93 ± 0.26 | 3.24 ± 0.08 | 4.07 ± 0.11 | 3.34 ± 0.22 |
| 7-8 Roll | -11.79 ± 5.44 | 2.08 ± 3.00 | -4.66 ± 1.07 | 14.92 ± 3.03 |
| 7-8 Shift | -0.20 ± 0.31 | 0.29 ± 0.17 | -1.04 ± 0.08 | -1.08 ± 0.13 |
| 7-8 Slide | -1.12 ± 0.58 | -0.76 ± 0.06 | 1.13 ± 0.03 | 0.06 ± 0.13 |
| 7-8 Tilt | 5.16 ± 1.81 | -1.32 ± 0.87 | 4.58 ± 0.66 | 5.73 ± 1.01 |
| 7-8 Twist | 27.36 ± 2.92 | 28.76 ± 1.99 | 33.64 ± 1.35 | 35.03 ± 1.60 |
| 8-17 Buckle | -0.61 ± 0.92 | -0.25 ± 0.57 | -2.19 ± 0.36 | 0.28 ± 1.44 |
| 8-17 Opening | -3.39 ± 3.28 | -1.70 ± 0.42 | -1.00 ± 0.85 | -0.63 ± 2.28 |
| 8-17 Propel | 2.51 ± 1.93 | -1.22 ± 0.43 | -4.19 ± 0.62 | 4.29 ± 3.06 |
| 8-17 Shear | -0.12 ± 0.11 | 0.02 ± 0.07 | 0.16 ± 0.03 | -0.02 ± 0.20 |
| 8-17 Stagger | 0.00 ± 0.03 | -0.01 ± 0.01 | -0.10 ± 0.01 | -0.09 ± 0.05 |
| 8-17 Stretch | -0.12 ± 0.06 | -0.22 ± 0.02 | -0.09 ± 0.02 | -0.11 ± 0.07 |
| 6-7 Dihedral | 52.61 ± 2.54 | 9.15 ± 5.03 | 35.60 ± 2.80 | 7.50 ± 3.16 |
